# Discrete wavelet transform-driven optimized deep learning-based framework for dyslexia detection using EEG signals

**DOI:** 10.3389/fninf.2026.1765088

**Published:** 2026-03-25

**Authors:** Tabassum Gull Jan, Sajad Mohammad Khan, Sajid Yousuf Bhat, Zaid Ahmad Wani, Syed Immamul Ansarullah, Sami Alshmrany, Shafat Khan

**Affiliations:** 1 Department of Computer Science, University of Kashmir, Srinagar, India; 2 Department of Psychiatry, IMHANS, GMC, Srinagar, India; 3 Department of Management Studies, University of Kashmir, Srinagar, India; 4 Faculty of Computer and Information Systems, Islamic University of Madinah, Madinah, Saudi Arabia; 5 Department of Computer Science, College of Computer Science, King Khalid University, Abha, Saudi Arabia

**Keywords:** deep neural network, DWT, dyslexia, MRMR, ReliefF, shallow neural network

## Abstract

**Purpose:**

Dyslexia is a prevalent neurodevelopmental disorder that impairs a children’s ability to reading, writing, and language processing despite normal cognitive skills. Early identification is vital for timely support and interventions in children with dyslexia. This study aimed to develop an efficient EEG-based pipeline for dyslexia detection using deep learning techniques, while providing a consistent evaluation protocol for fair comparison across models and prior approaches.

**Methods:**

EEG recordings were acquired from 51 participants (26: dyslexic and 25: non-dyslexic), aged 5–10 years, during cognitive task performance. These signals were processed, segmented, and decomposed into standard frequency bands (alpha, beta, delta, and theta) using the discrete wavelet transform to capture discriminative neural patterns. Filter-based feature selection techniques were applied before classification to optimize performance and reduce redundancy to identify the most informative features. These ranked and individual band-wise features were systematically evaluated with classical machine learning baselines (Decision Trees, SVM, k-NN, and ensemble learners) alongside the proposed deep neural networks. In addition, we benchmarked end-to-end raw-EEG deep learning baselines (1D-CNN, LSTM, and EEGNet) and re-implemented representative existing pipelines, all evaluated on our dataset using the same evaluation protocol.

**Results:**

The proposed compact deep neural network with four hidden layers achieved the best performance, reaching classification accuracy of 98.85%, outperforming all baseline models, raw-EEG deep learning baselines, and re-implemented approaches.

**Conclusion:**

These findings support the feasibility of DWT-driven EEG analysis combined with deep learning for more accurate and early dyslexia detection. The proposed approach holds promise as a non-invasive screening tool to support improved educational outcomes through early diagnosis and targeted intervention.

## 1 Introduction

Learning disability (LD) is a neurodevelopmental condition that can interfere with the brain’s ability to process and understand information. LD typically involves disrupting one or more essential cognitive functions such as reading, writing, or solving arithmetic problems (Fajariyanti et al., 2022). These can also occur alongside other neurological conditions, including attention deficit problem, language comprehension and processing difficulties, and behavioral issues. LD is commonly grouped into three main types: dyslexia, dysgraphia and dyscalculia. Dyslexia mainly affecting reading, dysgraphia affecting writing; and dyscalculia which involves difficulties with mathematical tasks. For some children, learning can be further complicated by additional sensory issues, such as auditory and visual processing deficits. These difficulties make them harder to interpret sounds or process visual information. Among these, dyslexia is the most prevalent, affecting approximately 5–17% of the population, and is most often associated with difficulties in phonological processing (Tamboer et al., 2016).

Dyslexic children often struggle with academic difficulties affecting their confidence. Such students experience low self-esteem and diminished motivation. Overtime, these struggles end up with frustration and even in some cases leads to depression. From this point of view, it must be emphasized that early diagnosis is crucial so that affected children can receive the support they need before developing entrenched academic and emotional problems.

In traditional settings, diagnosis of dyslexia is made through combination of psychological and educational assessments. These standardized tests are conducted by trained and expert professionals. The evaluation is performed after a child had started schooling, and behavioral signs of learning challenges faced by them are already evident. While the approach is very common out there, it has clear drawbacks. These procedures identify dyslexia only after the problems have emerged. Such tests rely largely on observable behaviors during screening rather than directly reflecting underlying cause of neurological differences. Consequently, there arises the need for methods that are objective and neurophysiologically based for the earlier and more reliable detection of dyslexia.

Recent developments in neuroimaging have advanced understanding of the role of brain function in learning disabilities which include dyslexia. A range of data modalities such as magnetic resonance imaging (MRI) (Zahia et al., 2020), functional MRI (f-MRI) (Sihvonen et al., 2021), structural MRI (sMRI) (Lr and Sudha Sadasivam, 2022), computed tomography (CT) (Wang et al., 2019), positron emission tomography (PET) (Paulesu et al., 1996), electroencephalography (EEG) (Zainuddin et al., 2022; Shirly and Jerritta, 2021), magnetoencephalography (MEG) (Mittag et al., 2022; Gallego-Molina et al., 2022) have been used widely by researchers to investigate the brain functionality associated with dyslexia. Among various modalities available to us, EEG is the most affordable, portable and scalable. Also, it has high temporal resolution which is great for capturing fast and dynamic brain responses. This makes it more suitable for large-scale screening studies typically involving children. EEG has been used by many researchers for Brain-Computer Interfaces (BCI) and classification systems for different types of neurological disorders. These include: autism (Xu et al., 2024), epilepsy (Lasefr et al., 2023), drowsiness (Chaabene et al., 2021), Parkinson’s (Aljalal et al., 2022), and Alzheimer’s disease (Safi and Safi, 2021). Its ability to capture dyslexia-related neural signatures has motivated various EEG-based ML and DL studies. Which include: Guhan Seshadri et al. (2023) reported a study which they conducted of an EEG-based deep learning framework for classifying children with learning disabilities. They segmented EEG signals and extracted features from alpha, beta, gamma, theta, and delta bands. Different feature selection methods were employed, with ReliefF algorithm attaining an accuracy of 95.8%. Manghirmalani et al. (2011) employed a learning vector quantization method based on soft computing for classifying learning-disabled children using a dataset of 160 normal and 80 LD participants. This method attained an accuracy of 91.8%. These learning-disabled children were further divided into three subtypes: dysgraphia, dyslexia, and dyscalculia. Al-Barhamtoshy and Motaweh (2017) used computational analysis to study brain signal patterns in the left and right hemispheres, whereas Mohamad et al. (2015) and Perera et al. (2018) studied patterns of neural activation in the course of writing and reading activities, and reported neural asymmetries between children with dyslexic and typically developing peers. Christodoulides et al. (2022) explored spectral and entropy-based features and highlighted their usefulness and potential as biomarkers for dyslexia identification. More recent works (Ortiz et al., 2020; Formoso et al., 2022) have integrated temporal and spatial EEG descriptors with machine learning (ML) models such as support vector machine (SVM), random forest (RF), convolutional neural network (CNN), and entropy-derived features, achieving significant and notable improvement in classification.

In addition to feature-based machine learning pipelines, recent studies have increasingly explored deep learning architectures that learn discriminative patterns directly from raw EEG or from time–frequency representations (Peng et al., 2022). Convolutional neural networks (CNNs) (Chaabene et al., 2021; Tawhid et al., 2024; Demir et al., 2021) have been widely adopted because they can learn data-driven temporal filters from epoched EEG and capture local time-domain patterns (Zhao et al., 2019). Depending on the representation, researchers have applied 1D-CNNs directly on raw EEG segments to capture local patterns in EEG epochs (Alkhrijah et al., 2025; Ileri et al., 2020) or 2D-CNNs on transformed time-frequency representations such as spectrograms or wavelet-based scalograms, enabling the network model to exploit both spectral and temporal information (Edderbali et al., 2023; Wang et al., 2023). Recurrent neural network (RNN) models such as long short-term memory (LSTM) (Hanafi et al., 2023; Li et al., 2022) and gated recurrent unit (GRU) (Liu et al., 2024) variants have also been adopted to model sequential dependencies across time samples, which may reflect sustained cognitive processing during task execution. Compact EEG-specific architectures have gained attention. In particular, EEGNet (Lawhern et al., 2018) like models are frequently used as strong baselines because they employ depth wise/separable operations to learn spatial filters across channels with relatively few parameters, which is advantageous for small EEG datasets.

More recently, attention, and transformer based models (Alkhurayyif and Sait, 2024; Song et al., 2023; Narsimha Reddy et al., 2024) have been investigated for EEG classification, motivated by their ability to model global dependencies. While reporting high performance gains, these models often require larger datasets or additional strategies such as strong augmentation, pretraining, or self-supervised learning to generalize reliably in subject-limited biomedical settings.

Although several EEG-based dyslexia detection studies report promising results, direct comparison across works remains challenging because datasets and experimental conditions differ substantially, including task design (resting-state vs. task-evoked), electrode montages, preprocessing choices (filtering and artifact handling), epoch length, and evaluation strategies. In particular, reported performance of deep learning methods varies widely across studies, and sample-level splits may inadvertently mix epochs from the same subject across training and testing, leading to inflated estimates. This motivates the need for consistent benchmarking under a unified experimental protocol when comparing feature-based and deep learning-based approaches. Therefore, in addition to proposing our optimized framework, we re-implemented representative published pipelines and evaluated them on our task-evoked dataset using the same preprocessing and validation protocol.

Based on the above literature, the following research gaps are identified:

1. *Limited systematic band-wise analysis:* Several studies use a subset of EEG bands or a single feature extraction path, making it unclear which bands and descriptors contribute most to dyslexia discrimination.
2. *Inconsistent feature selection evaluation:* Although feature selection is widely used, comparative evaluation of redundancy-aware (MrMr) and neighborhood-based (ReliefF) methods under the same dataset and validation setup is still limited.
3. *Insufficient unified benchmarking:* Many works report results using either classical machine learning or deep learning alone, without benchmarking both families under a unified preprocessing, feature set, and validation protocol.
4. *Reporting and reproducibility:* Several studies report only a single best score, while cross-fold variability and clarity of reporting (e.g., mean versus best-fold performance) are often insufficient, limiting fair comparisons across methods and datasets.

To address these gaps, we propose an optimized EEG-based framework that combines multi-band feature extraction with a dual feature-selection strategy (MrMr and ReliefF) and evaluates both ML and neural network-based models (shallow as well as deep) for early dyslexia detection.

The objectives of this study are to:

1. Build and validate a task-evoked EEG dataset for dyslexia screening by analyzing recordings from dyslexic and non-dyslexic participants acquired during learning-related cognitive activities.
2. Develop an end-to-end EEG screening pipeline that includes preprocessing and artifact handling, segmentation into 10-s non-overlapping epochs, and extraction of structured features suitable for classification.
3. Perform multi-band EEG characterization using wavelet decomposition, by applying db4 DWT (level-6) to obtain band-specific representations (delta, theta, alpha, beta), and conduct five-band ablation including gamma to examine the contribution of additional bands.
4. Optimize discriminative EEG features using dual feature selection, by comparing MrMr and ReliefF and evaluating Top-10, Top-20, and Top-30 feature subsets under both band-wise and fused feature settings.
5. Benchmark shallow, deep, and raw-EEG deep learning baselines under a unified evaluation protocol, by comparing the proposed efficient DNN against classical ML models, a shallow neural network, and end-to-end raw EEG baselines (1D-CNN, LSTM, EEGNet) using the same preprocessing and cross-validation setup to ensure fair comparison.

The novel contributions of this study are as follows:

- *Task-evoked EEG dataset:* We analyzed task-evoked EEG recordings from 51 participants (dyslexic and non-dyslexic groups) during cognitive activities designed to elicit learning-related neural responses.
- *End-to-end EEG pipeline for task-based screening:* We present a complete pipeline covering acquisition and preprocessing (including artifact handling), 10 s non-overlapping epoching (34 epochs/subject; 1,734 total epochs), and structured feature engineering for dyslexia detection.
- *DWT-driven band decomposition of EEG:* Each epoch is decomposed using db4 DWT at level 6 into delta, theta, alpha, beta bands (with a reported 5-band ablation to validate excluding gamma).
- *Dual feature-selection analysis with Top-k evaluation:* We systematically compare MrMr vs. ReliefF to study relevance-redundancy tradeoffs, and evaluate Top-10/20/30 feature subsets under band-wise and fused configurations.
- *Unified benchmarking with efficient deep model:* We propose a computationally efficient DNN and benchmark it against classical ML and a shallow NN, and additionally compare with end-to-end raw EEG baselines (1D-CNN, EEGNet, LSTM) and existing works under the same evaluation setting.

The article is structured as follows: section 2 details participants, experimental protocol, data collection, preprocessing, feature extraction, and feature selection procedures. Sections 2.7 and 2.8 describe the baseline machine learning classifiers and the proposed deep learning architecture used for classification, respectively. Section 2.9 details validation protocol, section 3 reports and discusses results, and section 4 presents ablation study and section 5 concludes the study with the Section 6 presenting limitations and directions for future work.

## 2 Materials and methods

**Figure 1:**
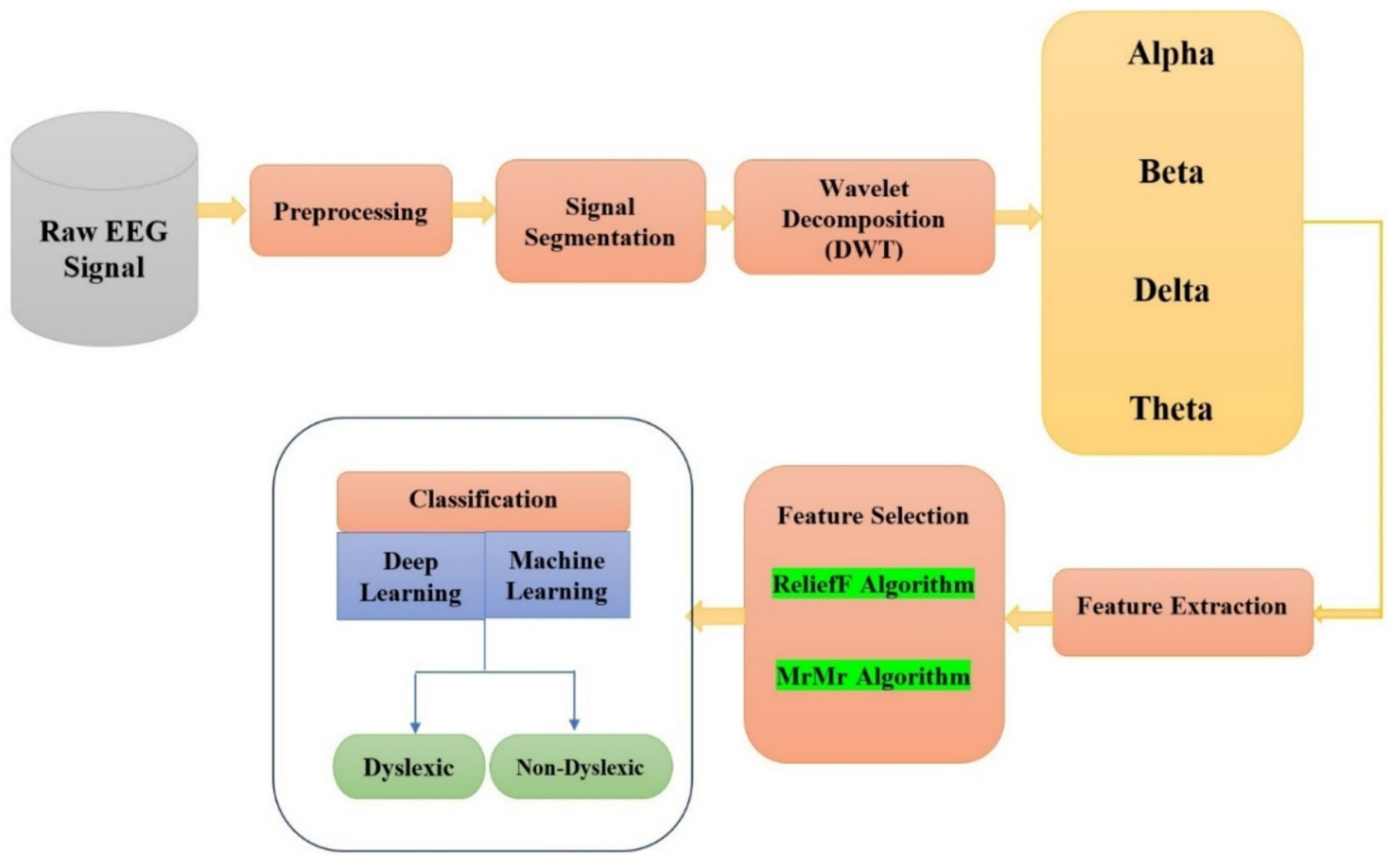
Illustration of the proposed EEG-based dyslexia detection pipeline, including raw task-evoked EEG signals, preprocessing, segmentation, DWT based sub-band decomposition, feature extraction, dual feature selection, and ML/DNN classification.

This section describes the general systematized approach, which encompasses the setup for EEG acquisition, the design of the screening tool, the collection of data, preprocessing, feature extraction and selection procedures. The classification was performed using features derived from the EEG recordings of both dyslexic and non-dyslexic children, employing both DL and ML approaches. Figure 1 shows a concise workflow that links these stages to final classification and evaluation.

This study is based on data from 51 participants residing in different parts of the Kashmir Valley with distribution given in Table 1. These participants were subjected to specific learning disability (SLD) evaluation using the SLD battery administered by qualified psychologists at the Institute of Mental Health and Neurosciences (IMHANS), Kashmir. Participants identified as having learning disability by SLD battery evaluation were then subsequently assessed by psychologists using the Dyslexia Assessment for Languages of India (DALI tool) in the English language. The age-appropriate DALI screening module was selected for each child (Junior Screening Tool (JST) (5–7 years); Middle Screening Tool (MST) (8–10 years)). In DALI screening, a total score is computed and compared against the prescribed cutoff score provided in the DALI tool. For English, the cutoff is ≥12 for JST and ≥23 for MST. Using this standardized scoring framework, children were labeled dyslexic when their DALI total score met or exceeded the corresponding cutoff for their module (JST/MST), and labeled non-dyslexic otherwise. All borderline cases were handled according to the standard criteria of the tool. These DALI-derived group labels were strictly verified by psychologists according to the predefined scoring rule and were used as the ground truth for all binary classification experiments conducted in this study. To minimize confounding influences on EEG patterns, children with known neurological disorders (e.g., epilepsy, traumatic brain injury), major sensory impairment (uncorrected vision/hearing problems), psychiatric illness, diagnosed comorbid neurodevelopmental disorders (e.g., Autism Spectrum Disorder (ASD) and Attention-Deficit/Hyperactivity Disorder (ADHD), developmental language disorder), or medications likely to affect EEG, or evidence of global cognitive impairment identified during clinical evaluation were not included in the dataset. The study was approved by the Board of Research Studies (BORS) at the University of Kashmir and by the Institutional Review Board (IRB) of Government Medical College (GMC), Srinagar (Ref. No: IRBGMC/CS 118). Before data collection, informed consent was obtained from the parents/guardians of all participating children. Each participant completed screening using a novel interactive application developed in Python, designed to measure time-based responses during tasks targeting visual, auditory, phonological, word recognition, picture naming, and working memory. EEG was recorded throughout the entire screening session, with a 5.6-min recording duration per participant. The combined behavioral and neurophysiological design enables the synchronized recording of EEG signals and the task performance.

**Table 1:** Participant summary.

Class	Number of subjects	Sex ratio (M/F)
Dyslexic	26	10/16
Non-dyslexic	25	4/21

**Figure 2:**
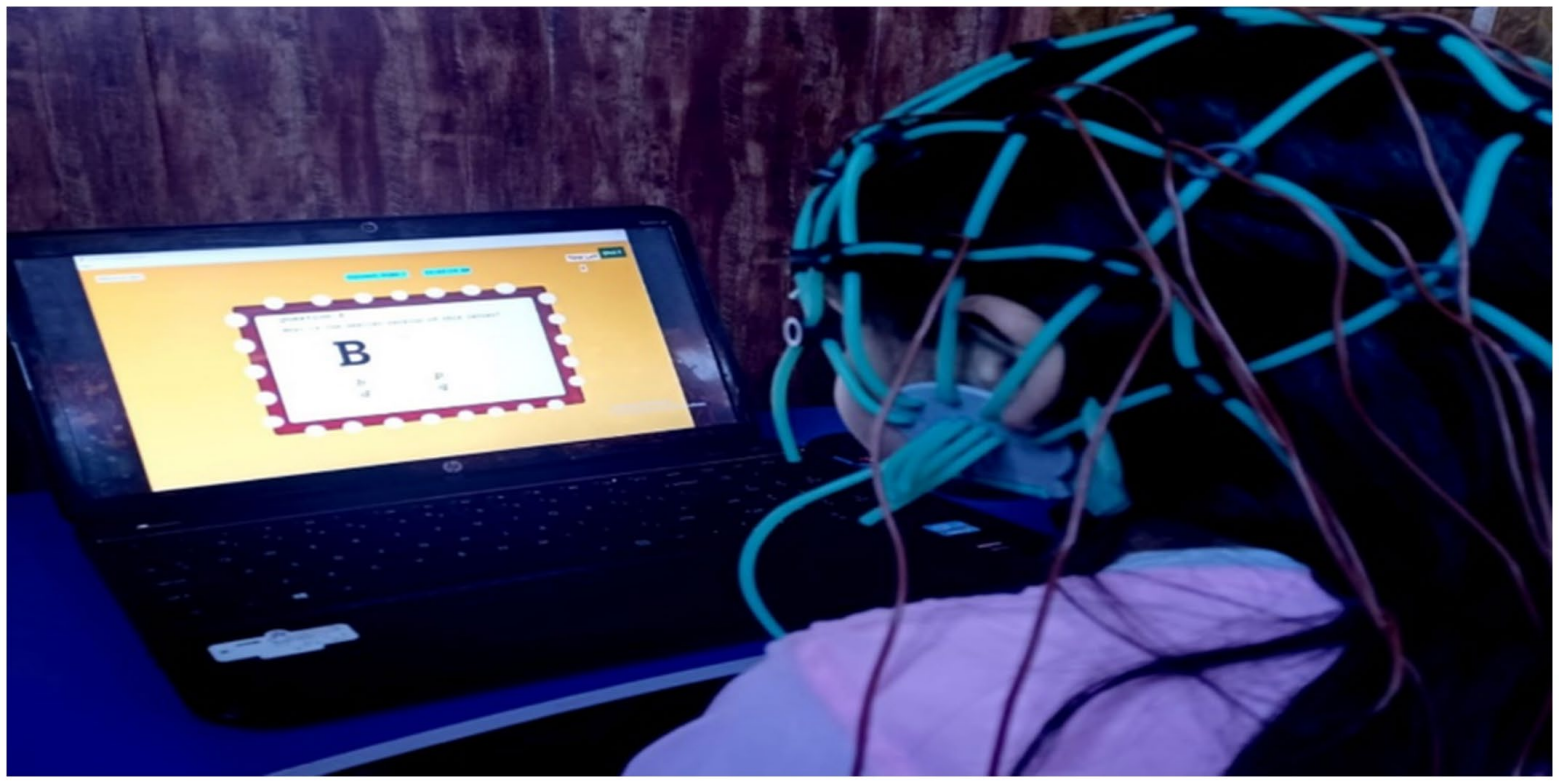
EEG recording environment during time-based screening tasks, illustrating the participants seating setup and acquisition conditions used for task-evoked EEG.

To support this design, we developed a Python-based screening application using Tkinter, OpenCV, and MNE-Python library. The application includes learning tasks that target domains associated with dyslexia. These include; learning areas such as reading and writing skills, phonological, visual memory and auditory processing, phoneme differentiation, rapid naming, working memory, arithmetic processing (addition and subtraction) and storytelling-related language abilities. A total of 17 questions were carefully designed by psychologists at IMHANS to target the specific areas of learning in which dyslexic students usually face difficulties. The questions were developed in an animated form, like a game, to include interactive screens for maximum student participation during screening. In addition, questions were designed in a time-limited manner, ensuring accurate measurement of responses during the allotted time for questions (20 s per question). Figure 2 shows how a participant is being screened. The questions in the screening tool, based on areas of learning, are described below, clearly explaining each task and area of learning assessed.

- Q1, Q4, Q5, and Q6: Focus on reading where different perspectives are checked, such as comparing letters and letter identification.
- Q6, Q15, and Q16 focused on writing where words identified were typed based on hearing or what they saw in an image.
- Q5, Q10: Focuses on phonological processing, where the first letter of the given word is to be identified, and rhyming word identification.
- Q3, Q7: Focus on phoneme differentiation, where directional and visually similar things are to be identified. It also focuses on matching the audio cues with the text.
- Q1, Q2, Q3, Q8, Q9, Q13, and Q16: Focused on contents related to visual memory, executive memory, working memory, picture naming, and letter identification.
- Q7, Q10, Q15, and Q17 focused on auditory processing, through listening-based rhyming identification, typing words from audio, story recall after video, and matching audio cues with text.
- Q11, Q12, and Q14: Focus on arithmetic processing, where problems are presented visually in animated form for addition and subtraction.
- Q17: Focuses on recalling the details of the story after watching it.

The dyslexic group included 26 individuals (16 females and 10 males), whereas the non-dyslexic group consisted of 25 individuals (21 females and four males). The participant summary is tabulated in Table 1, with both classes in the same age range of 5–10 years.

### 2.1 Data acquisition

The participants were seated in front of a computer monitor and EEG was recorded during the screening tasks using scalp electrodes. EEG was acquired using the RMS EEG Acquire system with wet Ag/AgCl electrodes positioned according to the international 10–20 electrode placement system. The recordings were obtained in a common-reference (unipolar) configuration with linked earlobe reference A1-A2 and a forehead ground (Fpz region) at a sampling frequency of 256 Hz. A total of 19 electrodes were recorded; for the final analysis only 16 channels were retained and the midline electrodes (Fz, Cz, and Pz), were excluded to reduce redundancy and simplify channel set. In our preliminary analysis, these midline channels showed lower variance and weaker discriminative power related to retained channels. So, removal of them ensured the coverage of all functionally important regions while minimizing redundancy and noise. Furthermore, they were removed because they had no functional relevance to the cognitive tasks included in the screening tool. The channels retained include: Fp1, Fp2, F3, F4, F7, F8, T3, T4, T5, T6, P3, P4, O1, O2, C3, C4. The raw EEG signals were saved in EDF (European data format) format and preprocessing included a band pass filter (0.1–70 Hz) to remove noise and muscle artifacts prior to further analysis. In addition to our proposed approach, we re-implemented representative published pipelines and evaluated them on this dataset under the respective evaluation protocol of each method.

### 2.2 Preprocessing

Preprocessing is a fundamental step used to remove artifacts and noise from EEG signals. The preprocessing pipeline involved data filtering using a moving average filter (window size = 9) to reduce high-frequency fluctuations and slow drifts. Physiological artifacts, such as muscle and eye movement artifacts (eye blinks), were removed using Independent Component Analysis (ICA) (Delorme and Makeig, 2004; Hyvärinen and Oja, 2000). It separates multichannel EEG signals into statistically independent components, enabling artifact-related components to be isolated.

These components associated with artifacts were visually identified using reproducible criteria based on their characteristic spatial distributions, power spectra, and topomaps. Eye blink components (ocular artifacts) exhibit vigorous frontal activity and low-frequency components, whereas muscle artifacts exhibit high-frequency noise localized to the posterior regions. These identified components were discarded, and artifact-free components were used to reconstruct the EEG signals. Hence, true neural oscillations are preserved while minimizing contamination from non-neural sources. Because the screening tasks involve visual/auditory stimulation and responses, recordings may also contain task-related artifacts (e.g., eye movements during visual tasks and facial/jaw muscle activity); these were mitigated by the same ICA-based procedure. For quality assurance during dataset preparation, the identified components were visually verified; however, the artifact identification criteria are objective and the preprocessing can be executed in an automated manner for real-world deployment. The preprocessing procedures are kept fixed across all experiments, including re-implemented baselines, to ensure fairness.

### 2.3 Segmentation

The continuous EEG signal of 5.6 min was segmented into non-overlapping windows of 10s duration for both classes, generating 34 segments per subject. A total of 1,734 epochs/samples were generated for all subjects, with 884 for the dyslexic class and 850 for the non-dyslexic class (Table 2). In this study, each epoch of 10s is treated as an individual sample for subsequent analysis.

**Table 2:** Sample distribution and feature vector dimensions after segmentation.

Total number of samples	Dyslexic samples	Non-dyslexic samples	Duration of segment	Segments per subject	Number of features	Number of features per epoch	Combined feature vector size
1734	884	850	10 s	34	10	160	(1,734,160)

### 2.4 Signal decomposition using DWT

In this study, Discrete Wavelet Transform (Subasi, 2007; Remeseiro and Bolon-Canedo, 2019; Zainuddin et al., 2019) was applied to decompose segmented EEG data of 10-s duration each into distinct frequency bands, enabling the analysis of both high- and low-frequency components associated with the cognitive tasks performed during screening, as shown in Table 3. DWT provides a time-frequency signal representation, which is necessary for non-stationary signals such as EEG. This helps break down the EEG signal into multiple bands with varying frequency ranges associated with specific neural or cognitive activities, as shown in Table 3. The Daubechies-4 (db4) db4 wavelet is common in EEG related studies because of its ability to analyze non-stationary. In this study, we chose db4 because of its balanced time and frequency localization trade-off. With four vanishing moments, db4 is particularly well suited for detecting transient and oscillatory oscillations (spikes and bursts) in EEG signals. Moreover, it effectively preserves signal morphology during decomposition without additional distortion or smoothing. These properties make db4 a reliable choice for EEG-based analysis and classification tasks. The DWT was performed at level 6, resulting in a series of coefficients corresponding to frequency bands as described in Table 3.

**Table 3:** Wavelet coefficients, EEG bands and related cognitive states.

Frequency band	Range (Hz)	Wavelet coefficient (level)	Associated cognitive/physiological state
Delta	0.5–4 Hz	A6 (Level 6)	Deep sleep, unconscious processes (Başar et al., 2001; Sun et al., 2023)
Theta	4–8 Hz	D6 (Level 6)	Drowsiness relaxed attention (Klimesch, 1999; Raufi and Longo, 2022)
Alpha	8–13 Hz	D5 (level 5)	Mental coordination (Raufi and Longo, 2022)
Beta	13–30 Hz	D4 (Level 4)	Active thinking (Ray and Cole, 1985; Klados et al., 2016)
Gamma	30–60 Hz	D3 (Level 3)	High-level information processing, cognitive functioning, perception, and consciousness (Rufener and Zaehle, 2021; Fries, 2005)

### 2.5 Feature extraction

According to existing research, various statistical and spectral features are commonly derived from EEG data for further investigation and analysis of brain disorders (Ortiz et al., 2020). We initially extracted features from four EEG bands (delta, theta, alpha and beta), which are commonly associated with reading and cognitive processing tasks and tend to be more stable in scalp EEG recordings (Mahmoodin et al., 2019). The gamma is considered to be more susceptible to high frequency noise and EMG contamination in pediatric EEG. So, gamma band was excluded from our primary pipeline. However, to verify this choice, we have also conducted a five-band ablation study and reported the results in results section. This study extracted 10 handcrafted features across four frequency bands for all 16 channels. Together, these features capture the essential characteristics of EEG signals, which have been frequently used in EEG studies to analyze cognitive and brain disorders (Guhan Seshadri et al., 2023). This approach resulted in 640 predictors per sample (10 features × 16 channels × 4 bands) supplied as inputs to the classifiers. For each decomposed frequency band, 10 statistical handcrafted descriptors were computed namely mean, median, variance, standard deviation, skewness, kurtosis, interquartile range (IQR), mean absolute deviation (MAD), root-mean-square (RMS), and entropy.

The statistical features were calculated for each decomposed band in the analysis as follows:

1.*Mean*: It gives the average amplitude of the wavelet coefficients, which shows the central tendency of the neural oscillations. It is represented by $\bar{x}$ as shown in Equation 1, where N is the total number of wavelet coefficients and $x_{i}$ represents coefficients at the *ith* level.

$$\bar{x}=\frac{1\;}{N}\sum_{i=1}^{N}x_{i}$$ (1)

2.*Median:* It provides a good estimate of the central tendency of the wavelet coefficients, and is more resistant to the effect of outliers than the mean. It is the middle value of the coefficients and shows the typical level of amplitude of the neural oscillations in the specified band.
3.*Variance:* Variance measures how far the decomposed wavelet coefficient sets measure from the mean, describing the degree of fluctuation of oscillatory amplitudes in the coefficients within a certain frequency band. It is computed by $\sigma^{2}$ as shown in Equation 2:

$$\sigma^{2}=\frac{1\;}{N}\sum_{i=1}^{N}\;{\left(x_{i}-\bar{x}\right)}^{2}$$ (2)

where N is the number of wavelet coefficients, $x_{i}$ is the *ith* coefficient, and $\bar{x}$ is the mean.

4.*Standard deviation*: The standard deviation is the square root of the variance and provides a more interpretable measure of spread in the same units as the coefficients. It is denoted by σ as shown in Equation 3 and is the square root of the variance:


$$\sigma =\sqrt{\frac{1\;}{N}\sum_{i=1}^{N}\;{\left(x_{i}-\bar{x}\right)}^{2}}$$ (3)

It describes the degree of consistency and variability of the neural oscillations in a given frequency band.

5.*Skewness:* Skewness describes the asymmetry of the coefficient distributions of a given decomposed wavelet coefficient set. It is represented by *Skewness(x)* and calculated as shown in Equation 4:

$$\text{Skewness}(x)=\frac{\frac{1\;}{N}\sum_{i=1}^{N}\;{\left(x_{i}-\bar{x}\right)}^{3}}{\sigma^{3}}$$ (4)

where N is the number of wavelet coefficients, $x_{i}$ is the *ith* coefficient, $\bar{x}$ is the mean, and *σ* is the standard deviation. More values being below the mean is indicated by positive skewness. This indicates a rightward tilt of the curve. More values being above the mean is indicated by negative skewness, which indicates a leftward tilt. Skewness measures the irregular shifts in the distribution which can mean that there is some atypical activity in the neurons. As for the distribution of the amplitude of a decomposed wavelet,

6.*Kurtosis*: As for the distribution of the amplitude of a decomposed wavelet, Kurtosis can tell us about the “tailedness,” a feature that captures large outlier values, as well as the presence of such extreme values. It is calculated as in Equation 5:

$$\text{Kurtosis}(x)=\frac{\frac{1\;}{N}\sum_{i=1}^{N}\;{\left(x_{i}-\bar{x}\right)}^{4}}{\sigma^{4}}$$ (5)

where N is the number of wavelet coefficients, $x_{i}$ is the *ith* coefficient, $\bar{x}$ is the mean, and *σ* is the standard deviation. A more even and flat distribution is indicated by low kurtosis. This also indicates that the configuration has large deviations in the neural oscillations.

7.*Interquartile Range (IQR):* It reports the range of the central 50% of amplitudes in a decomposed wavelet set which reduces the effect of extreme values. As in Equation 6:

IQR=Q3−Q1 (6)

where Q1 and Q3 are the 25th and 75th percentiles, respectively.

8.*Mean Absolute Deviation (MAD):* It measures the average distance between each amplitude value in relation to the mean of a decomposed wavelet coefficient set. It provides a robust measure of variability that is less sensitive to extreme values. It is calculated as shown in Equation 7:

$$\mathrm{MAD}=\frac{1}{N}\sum_{i=0}^{n}|x_{i}-x_{i}|$$ (7)

where N is the number of wavelet coefficients, $x_{i}$ is the *ith* coefficient, $\bar{x}$ is the mean, and *σ* is the standard deviation.

9.*Root Mean Square (RMS):* This is a measure of the total amplitude variation within a decomposed wavelet coefficient set which in turn presents a picture of the total signal strength. It is calculated as shown in Equation 8:

$$\mathrm{RMS}=\sqrt{\frac{1}{N}\sum_{i=0}^{n}{x_{i}}^{2}}$$ (8)

where N is the number of wavelet coefficients, $x_{i}$ is the *ith* coefficient.

10.*Entropy:* Entropy is a measure of the uncertainty present in a signal. It measures complexity of the coefficient distribution and is commonly used as an indicator of signal irregularity. Mathematically, entropy *E_n_* is calculated using the following Equation 9:

$$E_{n}=-\sum P(x_{n})\log(P(x_{n}))\;$$ (9)

where $P(x_{n})$ denotes the probability distribution of the signal values $x_{n}$ . Larger entropy values indicate greater complexity and variability in neural activity.

### 2.6 Feature selection techniques

From a given set of features, classifiers perform better if only compact set of informative features are retained. Feature Selection methods aim to identify features that exhibit strong discriminative power by reducing dataset dimensionality. Such methods increase the stability of classification models at the cost of reducing computational demands. In addition to this, a large set of redundant features increases the likelihood of prolonged training and overfitting. Numerous feature selection algorithms have been used in the literature, such as filter-based, wrapper-based, embedded, hybrid, and ensemble methods (Lakretz et al., 2015; Jothi Prabha and Bhargavi, 2020). In this study, we opted for filter-based methods owing to their classifier independence and computational speed. For each decomposed band of the EEG signal sample, 640 predictors were extracted. The comprehensive representation of the signal features pertains to combining features from all bands (feature sets obtained after combining all band features: 10 features × 4 bands × 16 channels = 640 features). To reduce feature dimensionality and retain only discriminative features, two filter-based feature selection methods were used: ReliefF and MrMr. We have used MrMr to select features with high class relevance while minimizing inter-feature redundancy, and ReliefF to prioritize features that best separate neighboring samples across classes, capturing non-linear and local interactions.

#### 2.6.1 Minimum redundancy maximum relevance

The MrMr feature selection approach balances the relevance of each feature with the target variable and minimizes the redundancy among features (Rufener and Zaehle, 2021; Fries, 2005). This approach selects features with a higher correlation with the target variable and a low correlation with other features. This metric relies solely based on calculating mutual information between features and target variables. The MrMr aims to select feature subset S containing q features from full feature set $x_{i}$ , that have strong associations with the target class C. To maximize relevance, the mean mutual information between each feature $x_{i}$ in the feature subset and class label C, as shown in Equation 10:


$$I(X:Y)=\sum_{x,y}p(x,y)\log\frac{p(x,y)}{p(x)\;p(y)}$$ (10)

The Maximum Relevance criterion, which calculates the highest MI value, is given by Equation 11:


$$\max U(S,C),\;\;U=\frac{1}{S}\sum_{x_{i}\in S}I\;(x_{i};C)$$ (11)

where *C* is the target label, and I( $x_{i}$ ; C) is the mutual information between feature $x_{i}$ and the target class *C*.

An optimal feature subset has a low redundancy measure, meaning features are as unique as possible. To minimize redundancy, the feature that shared the least MI with the rest features in the set is retained, as shown in Equation 12:


$$\min V(S),\;\;\;\;V=\frac{1}{{|S|}^{2}}\sum_{x_{i},x_{j}\epsilon \;S}I(x_{i};x_{j})$$ (12)

where ∣S∣ is the subset of features, *I (* $x_{i}$ ; $x_{j}$ *)* and represents the mutual information between features $x_{i}$ and $x_{j}$ .

#### 2.6.2 ReliefF algorithm

The ReliefF algorithm, a filter-based feature selection method, is an extension of the Relief algorithm introduced by researchers in Lakretz et al. (2015) and Jothi Prabha and Bhargavi (2020). This algorithm assigns weights to each feature by randomly choosing a sample “R” from the training points and identifying how their nearest same class and different-class neighbors differ. This process is repeated *m* times to compute feature weights. A feature is more useful for classification if it receives a higher weight. The computational cost increases approximately in proportion to the feature count *n* and the sampling rate *z*, making it highly efficient. The original Relief algorithm only supported binary classification and was limited in handling missing data. ReliefF (Guhan Seshadri et al., 2023; Urbanowicz et al., 2018) assigns weights to features by randomly selecting a sample R from the training set and adjusting weights based on k-nearest hits and k-nearest misses from the target and other classes, respectively. By iterating this process z times, the final feature weights are determined. Algorithm 1 shown below provides the detailed outline of ReliefF algorithm.:

ALGORITHM 1 .ReliefF Feature Weighting for Informative Feature Selection.

1. Initialize all feature weights to 0.
2. For each of the *m* iterations:
  - Randomly select an instance R from the training set.
  - Identify the k-nearest neighbors from the same-class (hits) and different-classes (misses).
3. Update feature weights based on the differences between R and its nearest hits and misses:
  - Increase the weight if the feature is similar for hits.
  - Decrease the weight if the feature is similar for misses.
4. Output the final weights, where higher weights indicate more informative features.

This process yields a vector of feature weights, allowing features with higher weights to be considered more relevant for classification.

These techniques rank features based on their relevance to classification tasks. The “Top 10, Top 20, and Top 30” most relevant features were selected, representing the highest-ranked features from these methods. Each feature selected is aligned to a particular frequency band, EEG channel, and statistical metric. For example, the Top 10 features refer to the 10 highest-scoring features in the ranking. Similarly, the Top 20 and Top 30 were considered for the analysis. In this study we used selected feature subsets as input for many machine learning baselines and deep learning models.

The selected features were evaluated under multiple configurations which included band wise features, combined or fused features (4- band as well as 5-band), and refined feature subsets (Top 10, Top 20, and Top 30) which we chose using ReliefF and MrMr separately. This design allowed us to conduct a systematic assessment of classification performance across different ML and DL methodologies, guaranteeing a comprehensive analysis for each of the implemented feature selection strategies.

### 2.7 Classification using machine learning

In this section, we detail various ML models applied for classification of dyslexia using EEG signals. Initially, features were derived from 4 frequency bands, namely alpha, beta, theta, and delta as detailed in Table 3, to distinguish between dyslexic and non-dyslexic classes. Each sub-band contained 160 features (10 features across all EEG channels) per EEG signal sample, resulting in a feature matrix of size (160 × 884 × 1) {features × samples × class} for the dyslexic class and (160 × 850 × 1) for the non-dyslexic class. Next, features from all subbands were combined and fused to form a complete set of (640 × 1734 × 2) to classify dyslexic and non-dyslexic groups using machine learning. The models were trained on both the full feature set of 640 features and a subset of highly relevant features (Top 10, Top 20, and Top 30), selected using the filter-based (MrMr and ReliefF). After features are selected, the processed EEG features were utilized to train the ML classifiers, and the performance was assessed using stratified 10-fold cross-validation. The ML classifiers employed to distinguish dyslexic and non-dyslexic classes include Decision Trees (DT) (Laine et al., 2000), k-nearest neighbors (kNN) (Prabha et al., 2019), SVM (Gallego-Molina et al., 2022), ensemble methods (Ribeiro et al., 2022; Jan and Khan, 2023), linear discriminant analysis (LDA) (Safi and Safi, 2021; Jothi Prabha and Bhargavi, 2020), Logistic Regression (LR) (Gallego-Molina et al., 2022; Che et al., 2017), and their variants.

### 2.8 Classification using deep learning

**Figure 3:**
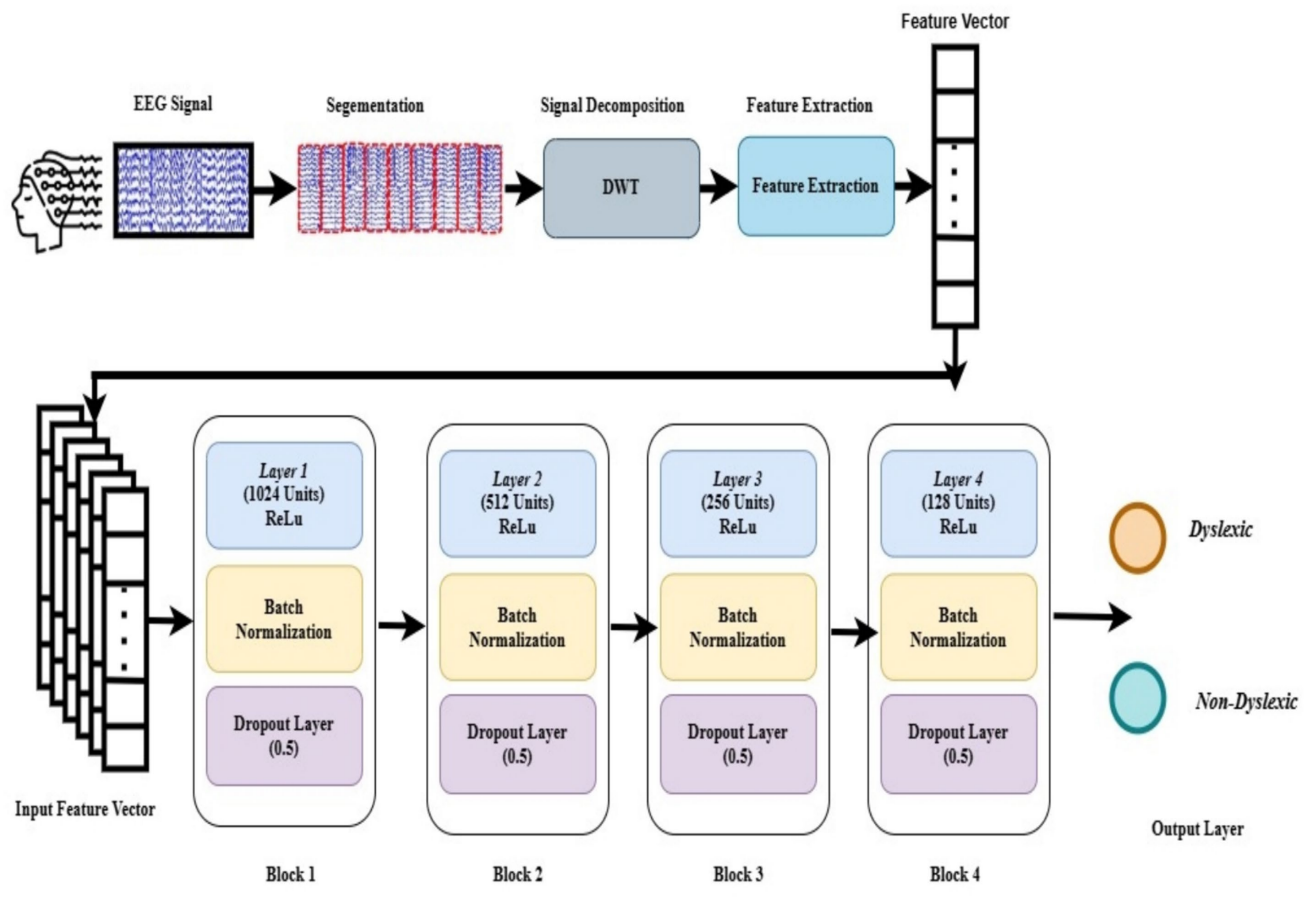
Architecture of proposed deep neural network, showing the sequence of fully connected layers, activation functions, and regularization components within each block.

Neural networks are broadly categorized as shallow or deep based on the number of hidden layers. Shallow networks (SNN) generally have a maximum of two hidden layers, while deep neural networks (DNNs) have multiple layers, usually greater than two. These architectures exhibit varied performances depending on the task, making it essential to select an appropriate depth when designing models (Opěla et al., 2022). Shallow networks are well-suited for tasks with low-dimensional data because of their ability to learn dominant features (Bejani and Ghatee, 2021) independently. In contrast, DNNs are better equipped to handle complex tasks involving large and high-dimensional datasets (Bejani and Ghatee, 2021). This study compares existing shallow neural networks and the proposed deep neural networks for classification. The advanced shallow model used in this study consisted of a feature input layer, three fully connected (FC) layers. The size of the feature input layer equals the feature vector dimension, which is 640 for combined feature set, 160 for individual bands and becomes k when Top-k feature selection is applied; this input feature vector is independent of the number of neurons in the hidden layers. Each layer incorporated batch normalization (BN) and rectified linear unit (ReLU) activation, with dropout layer (rate = 0.5) for regularization, and a final softmax output layer for classification. This advanced Shallow Neural network corresponds to Block 2, Block 3, and Block 4 of the proposed architecture, as illustrated in Figure 3. The proposed deep neural network (DNN) extends this design by including four FC layers with ReLU activation, BN, dropout (0.5), followed by dense layer, and a softmax output layer (Figure 3). The hyperparameters used to train the proposed DNN are listed in Table 4.

**Table 4:** Hyperparameters for the models.

Batch size	Optimizer	Learning rate	Max epochs
16	Nadam	0.001	30

To further compare the proposed DWT feature-based model with end-to-end learning, we evaluated raw EEG baselines (1D-CNN, EEGNet and LSTM) trained directly on the preprocessed 10-s EEG epochs at 256 Hz sampling frequency with 16 channels resulting into1734 epochs from 51 subjects. The 1D-CNN baseline comprised three Conv1D blocks (32/64/128 filters, kernel size 7) with BN, ReLU activation, max pooling, and dropout, followed by global average pooling (GAP) and a sigmoid output. The LSTM baseline down sampled the time axis using Conv1D and pooling before an LSTM (32) layer to reduce runtime. EEGNet was implemented using temporal convolution, depth wise spatial convolution (depth multiplier *D* = 2), separable convolution, average pooling, dropout, and a sigmoid output. All raw baselines were evaluated using the same cross-validation protocol as the proposed method. The detailed baseline architectures and their training details are provided in Supplementary material.

### 2.9 Validation protocol

The model performance was assessed using segment-level stratified 10-fold cross-validation, ensuring balanced representation of EEG segments of the two classes across each fold. Since, segmentation produces multiple correlated epochs per participant, epochs from the same child may appear in different folds under this evaluation strategy; therefore, the reported results should be interpreted as epoch-level internal validation and may represent an optimistic estimate of generalization to unseen subjects. Furthermore, the BN and dropout of 0.5 with an early stopping criterion during training helped the model to prevent overtraining and enhance generalization.

### 2.10 Performance metrics

This section presents a detailed overview of different performance metrices used to evaluate the effectiveness of the proposed classification model. Several standard performance metrices were calculated including accuracy, precision, recall, F1-Score and ROC Curve to evaluate the performance of the proposed DNN.

## 3 Results and discussion

All experimental procedures, including the development and execution of the ML and proposed DNN were implemented using the Python programming language (Python 3.9). The computations were performed on a NVIDIA DGX A100 AI-Server hosted at the Department of Computer Science, University of Kashmir, Srinagar. The server has 320 GB GPU Memory, 3.4GHz Dual AMD Rome 7,742 CPU, 1 TB System Memory, and 15 TB disk space. This section presents the experimental results obtained using the baseline machine learning classifiers and the proposed deep neural network. We report performance for (i) band-wise feature sets (delta, theta, alpha, beta, and gamma) and (ii) the combined multi-band feature representation. In addition, we analyze the impact of feature selection by evaluating subsets generated MrMr and ReliefF (Top 10, Top 20, and Top 30).

### 3.1 Performance on machine learning classifiers using band-wise features

Table 5 reports the performance of different ML classifiers across various frequency bands. In the different classification experiments, each of the individual EEG frequency bands and the overall multi-band feature set showed varying levels of performance. Overall, the advanced ensemble methods consistently obtained better performance than simpler single classifiers, indicating that combining multiple learners provides a more robust and reliable decision boundary for the EEG-based features.

**Table 5:** Classifier accuracies across different frequency bands.

Classifier	Accuracy (%)				
	Alpha band	Beta band	Delta band	Theta band	Combined
Fine tree	76.16338	75.4182	72.1331	78.2928	92.5148
Medium tree	76.96798	80.3731	78.2968	80.4246	94.1273
Coarse tree	80.54182	80.3687	77.4291	78.9868	93.3214
LDA	80.13255	73.0008	86.5893	82.6131	87.851
QDA	66.83709	69.1396	62.4656	64.2482	83.0171
Logistic regression	59.35486	63.7346	53.7197	55.3834	87.7932
Gaussian naive Bayes	60.85111	59.4671	60.394	72.4171	63.7861
Linear SVM	60.50595	64.7711	54.2894	54.9259	84.4565
Quadratic SVM	58.77982	60.3349	58.7792	59.2937	82.445
Cubic SVM	58.9519	60.2807	59.2399	61.1375	71.6271
Fine Gaussian SVM	60.39001	63.3842	60.9096	61.0777	89.1163
Medium Gaussian SVM	54.33958	54.3977	51.4056	53.2463	89.0589
Coarse Gaussian SVM	51.86532	52.9593	51.1179	51.1179	53.4768
Fine KNN (*k* = 3)	58.14464	59.1207	58.3214	60.6734	69.8904
Medium KNN (*k* = 10)	58.26291	58.4393	59.4133	60.5641	68.338
Coarse KNN (*k* = 100)	50.89562	54.114	55.0365	56.2484	72.7075
Cosine KNN (*k* = 10)	55.6664	56.6481	54.0575	52.9609	77.5487
Cubic KNN (*k* = 10)	57.11149	56.4218	56.8238	58.7768	65.288
Weighted KNN (*k* = 20)	56.36004	57.1125	60.3359	61.5976	67.2992
Ensemble boosted trees	81.86599	81.9205	75.4159	81.0006	96.4301
Ensemble bagged trees	85.83516	86.2414	84.9748	85.8953	96.0285
ESD	86.9301	88.0812	81.8623	85.6611	96.2012
EBT-RUS	86.18331	88.2533	81.8607	85.7202	96.0856

The Alpha band shows that Ensemble Subspace Discriminant (ESD) achieved the highest accuracy of 86.93%, while Ensemble Bagged Trees attained 85.83%, a close second. Ensemble Boosted Trees with RUS (EBT-RUS) and Ensemble Boosted Trees are other sophisticated techniques that had fairly high accuracy of 86.18 and 81.87%, respectively.

In the beta band, Ensemble Boosted Trees with RUS attained the best accuracy of 88.25%, narrowly ahead of the ESD which reached 88.08%. Ensemble Bagged Trees also performed well, achieving an accuracy of 86.24%. Among the traditional ML classifiers, the Medium Tree showed comparatively stronger performance (80.37%) than most other traditional methods, but still remained below the ensemble-based approaches.

For the delta band, Ensemble Bagged Trees again produced strong performance, reaching an accuracy of 84.97%, followed by ESD at 81.86%. The Medium Tree, maintained competitive performance in this band as well (accuracy = 78.29%), suggesting that delta-band features carry useful discriminative structure even for simpler models.

These reports present that delta and theta bands dominate in EEG based dyslexia detection. This is due to the role of low frequency oscillations in speech processing and syllabic analysis (Lizarazu et al., 2019) and phonological processing (Power et al., 2013), which are core issues in individuals with dyslexia. Also, EEG-related studies have reported increased theta activity in individuals with dyslexia (Başar et al., 2001) and altered phase synchrony, power, and temporal sampling during reading or auditory tasks (Lizarazu et al., 2019). Thus, the ML classifiers performed best in these bands as is expected in terms of neural markers associated with dyslexia.

Also, in theta band, EBT-RUS attained an accuracy of 85.72%, closely followed by Ensemble Bagged Trees at 85.90% and ESD at 85.66%. The Medium Tree, also performed well, achieving an accuracy of 80.42%.

When using the multiband combined feature set, the Ensemble Boosted Trees performed best a with accuracy of 96.43%, very close behind were the results from the EBT and ESD which reported 96.03 and 96.20%, respectively. Also, tree-based methods also performed well on the combined feature set with Medium Tree reporting an accuracy of 94.13%.

Overall, in most of the cases, advanced ensemble techniques proved superior to the traditional classifiers for all the different feature sets, especially for ESD, Ensemble Bagged Trees, and EBT-RUS. The best overall performance among the different feature sets was obtained with the combined feature set highlighting the gain from using various features in attaining optimal classification. While all the different feature bands employed individually showed that the tree-based methods were able to compete, ensemble methods for the combined features proved to be more effective and advanced in this domain.

#### 3.1.1 Justification

The results reported in Table 5 are explained by the inherent strengths of the ensemble learning. Across all the experiments, ensemble classifiers tend to give the most reliable performance compared with simpler single models. This is likely because ensemble models combine decisions from multiple learners, reducing the variance and bias of models. The advantage of these methods is clear in alpha and beta bands, where task-related cognitive patterns are more pronounced. Even in bands that tend to be less individually discriminative (such as delta and theta), ensemble methods maintained competitive performance. The best overall results (in terms of accuracy) came from using combined multi-band feature set, which suggests that fusing various frequency bands together makes feature subsets enriched with more information than feature subsets belonging to single band alone. Infact each band represents different aspects of neural activity, therefore combining them presents a broader and more useful representation for classification. Some individual tree-based models performed reasonably well in particular bands, but their performance tended to fluctuate more from one setting to another. Ensemble models on the other hand, were generally more consistent. That kind of stability is important when working with small noisy EEG datasets where number of subjects is limited.

### 3.2 Evaluation of machine learning classifiers with feature selection

The classification results presented in Table 6 show a clear pattern that performance depends not only on feature-size but also on which feature selection method is used and how well it matches the classifier. For the Top 10 MrMr features, the best results came from ensemble methods, with accuracy of Ensemble Bagged Trees (64.99%), ESD (64.48%), and Ensemble Boosted Trees (64.25%). The Coarse Tree and QDA in comparison produced moderate results with accuracies of 58.38 and 58.89%, respectively. On the other hand, a different trend appeared with ReliefF-selected feature subset. The selected features strongly benefited LDA achieving much higher accuracy of 81.29%, well ahead of other classifiers such as Fine Gaussian SVM (59.58%) and Fine KNN (57.97%). Hence, the feature space produced by these selected features is suitable for linear separation. When the feature subset was expanded to Top 20 MrMr, ensemble methods continued to dominate. The best accuracy was attained by ESD (71.39%), followed by EBT-RUS (71.33%) and Ensemble Bagged Trees (69.89%). Among conventional classifiers, LDA still remained competitive at (67.88%), and the Medium Tree also performed well (66.78%). For Top 20 ReliefF-selected features, LDA again remained the best-performing classifier with an accuracy of 81.24%. In comparison, ensemble models showed a noticeable drop in performance in this setting, with Ensemble Bagged Trees reaching only 56.76% accuracy. Hence, feature space produced by ReliefF features align better with simple linear classifiers than with ensemble learners.

**Table 6:** Classifier accuracy across different feature selection techniques.

Classifier	Accuracy (%)					
	MrMr			ReliefF		
	Top10	Top 20	Top 30	Top 10	Top 20	Top 30
Fine tree	56.0106	67.2401	66.7813	60.6149	60.6149	60.8498
Medium tree	56.8746	66.7816	66.6677	59.6937	59.7515	60.3893
Coarse tree	58.3755	66.378	66.6653	59.409	59.0632	59.2379
LDA	52.2131	67.8752	70.296	81.2939	81.2365	85.0299
QDA	58.8941	69.4343	63.445	52.2175	51.7567	54.6861
LR	52.1557	52.046	52.1042	56.2454	56.2454	55.8996
GNB	58.8934	60.0462	65.8039	58.6625	58.6625	59.0107
Linear SVM	52.156	52.2155	52.2743	57.6261	57.6261	57.5686
Quadratic SVM	58.3765	59.123	59.123	55.7239	55.5511	55.5521
Cubic SVM	52.5018	50.4269	50.4269	55.091	55.0336	55.2073
Fine Gaussian SVM	59.0658	58.3752	58.3752	59.5801	59.3499	59.5851
Medium Gaussian SVM	55.5448	54.8	53.9366	57.3952	57.9696	58.9562
Coarse Gaussian SVM	55.5448	54.7425	54.7425	57.3952	57.3952	57.8623
Fine KNN (*k* = 3)	56.3019	55.7797	55.7797	57.9732	57.9732	58.6074
Medium KNN (*k* = 10)	55.9524	56.3574	56.3574	55.2648	55.2648	56.1348
Coarse KNN (*k* = 100)	56.0677	56.1295	56.1295	56.36	56.36	56.075
Cosine KNN (*k* = 10)	55.9521	54.3392	54.3392	53.9462	53.9462	53.4317
Cubic KNN (*k* = 10)	55.8401	55.0302	55.0302	54.7475	54.7475	56.2504
Weighted KNN (*k* = 20)	57.3939	56.7567	56.7567	57.453	57.453	58.2028
Ensemble boosted trees	64.2462	70.8707	72.1929	59.0605	59.0605	57.742
Ensemble bagged trees	64.993	69.893	72.7124	56.7022	56.761	57.6895
ESD	64.4755	71.3856	73.4024	56.1255	57.163	57.5145
EBT- RUS	63.8971	71.3268	73.5147	56.5879	56.0102	57.1713

With regard to Top 30 MrMr-selected features, ensemble classifiers again delivered strong performance with ESD achieved an accuracy of 73.40%, while EBT-RUS reached (73.51%) and Ensemble Bagged Trees obtained (72.71%). Also, traditional classifiers like LDA performed quite well in this setting reaching 70.30%. This suggests that feature subsets with increased size in MrMr retained useful discriminative structure even for simpler models as well. The trend was different for ReliefF. Under Top 30 ReliefF setting, ensemble methods performed relatively poor with ESD and Ensemble Bagged Trees achieving accuracies of 57.51 and 57.68%, respectively.

Overall, the results showed a clear difference between the two feature selection techniques. MrMr feature selection tends to work best with ensemble methods, especially when feature subset size is increased. ReliefF feature selection on the other hand consistently benefited LDA, and it performed well with Top 30 features. From practical perspective, this suggests that ReliefF is capturing subtle but stable patterns that can be separated well with a linear boundary, which may reflect dyslexia-related processing differences. Taken together, these findings indicate that ReliefF-selected features are more compatible and pair better with simpler linear classifiers, whereas MrMr-selected features are aligned better with the higher-capacity decision mechanisms of ensemble methods.

#### 3.2.1 Justification

The difference in performance across the different experiments is presented in Table 6. The table signifies the performance dependence on interaction of feature selection technique with the strengths of different classifiers. MrMr feature selection technique consistently favored ensemble methods across all feature subset sizes. ESD and Ensemble Bagged Trees produced consistently good results with performance increase with increase in feature subset size. This is attributed to the diverse set of moderately informative features used in ensemble methods with their aggregation behavior reducing overfitting and improves generalizability. However, ReliefF-selected features better alignment with linear models (such as LDA) with the best overall performance and an accuracy of 85.03% using Top 30 ReliefF features. These patterns observed in MrMr feature selection method tends to reduce redundancy while preserving features that are globally informative and relevant. On the contrary, ReliefF prioritizes neighborhood-based relevance and local separability which produces feature spaces that are closer to linearly separable, thus well matched to LDA. These findings highlight the importance of choosing feature selection techniques and classifiers in order to maximize its performance.

### 3.3 Evaluation of deep learning models with band-specific features and feature selection approach

The results presented in Tables 7, 8 indicate that ReliefF-selected feature subsets achieve the best classification performance across both the Advanced shallow neural network and proposed DNN with increase in feature subset size. The role of delta features in cognitive processes is evident through the highest performance in both networks, demonstrating a maximum accuracy of 95.40% (Advanced SNN, Table 7) and 95.95% (Proposed DNN, Table 8). Theta band features also showed a strong performance with a maximum accuracy of 94.79%, particularly with the DNN. Overall, the combined feature set performed well but the reported performance still remained below the delta and theta bands when used in separation. This indicates that focusing on the most informative bands followed by application of appropriate feature selection method can be more effective in dyslexia classification instead of using the full complete feature set.

**Table 7:** Performance of advanced SNN using four frequency bands.

Feature type	Input size	Dataset	Advanced shallow neural network				
			Accuracy (%)	Precision (%)	Recall (%)	F1 score (%)	Roc
30 ReliefF (4 bands)	Top 30	Average	95.39	95.52	95.39	95.39	0.9923
		Maximum	98.26	98.27	98.27	98.27	0.9993
20 ReliefF (4 bands)	Top 20	Average	95.34	95.41	95.34	95.33	0.9925
		Maximum	98.28	98.33	98.28	98.27	0.9993
10 ReliefF (4 bands)	Top 10	Average	95.34	95.44	95.34	95.33	0.9918
		Maximum	97.12	97.18	97.12	97.12	0.9984
30 MrMr (4 bands)	Top 30	Average	82.56	82.76	82.56	82.53	0.9170
		Maximum	87.28	87.28	87.28	87.28	0.9368
20 MrMr (4 bands)	Top 20	Average	77.38	77.70	77.38	77.29	0.8702
		Maximum	81.50	82.87	81.50	81.27	0.8970
10 MrMr (4 bands)	Top 10	Average	60.74	61.21	60.74	60.00	0.6380
		Maximum	67.63	68.70	67.63	67.02	0.7368
Alpha Features	160	Average	85.67	86.09	85.66	85.61	0.9401
		Maximum	89.66	89.72	89.66	89.64	0.9636
Beta Features	160	Average	80.08	80.73	80.08	79.94	0.8742
		Maximum	82.65	83.10	82.65	82.65	0.9270
Theta Features	160	Average	89.81	89.95	89.81	89.80	0.9630
		Maximum	94.79	94.94	94.79	94.79	0.9741
Delta Features	160	Average	93.49	93.57	93.49	93.49	0.9821
		Maximum	95.40	95.49	95.40	95.39	0.9915
Combined Features (4 bands)	640	Average	93.38	93.47	93.38	93.37	0.9832
		Maximum	95.97	95.98	95.98	95.97	0.9939

**Table 8:** Performance results of the proposed DNN using only for frequency bands (δ, θ, α, β).

Feature type	Input size	Dataset	Proposed deep neural network				
			Accuracy (%)	Precision (%)	Recall (%)	F1 score (%)	Roc
Alpha features	160	Average	85.90	86.37	85.89	85.82	0.9375
		Maximum	90.23	90.35	90.23	90.22	0.9642
Beta features	160	Average	80.14	81.70	80.14	79.93	0.8724
		Maximum	84.48	84.62	84.48	84.48	0.9435
Theta features	160	Average	89.23	89.36	89.24	89.22	0.9621
		Maximum	93.68	93.73	93.68	93.67	0.9765
Delta features	160	Average	92.79	92.88	92.74	92.74	0.9803
		Maximum	95.95	96.25	95.95	95.94	0.9957
Combined features (4 bands)	640	Average	93.08	93.22	93.08	93.08	0.9834
		Maximum	96.55	96.57	96.55	96.55	0.9953
30 ReliefF (4 bands)	Top 30	Average	95.74	95.77	95.62	95.62	0.9924
		Maximum	97.68	97.69	97.69	97.69	0.9995
20 ReliefF (4 bands)	Top 20	Average	95.27	95.40	95.28	95.27	0.9930
		Maximum	98.85	98.88	98.85	98.85	0.9984
10 ReliefF (4 bands)	Top 10	Average	95.10	95.26	95.10	95.10	0.9916
		Maximum	97.70	97.73	97.70	97.70	0.9992
30 MrMr (4 bands)	Top 30	Average	82.27	82.50	82.26	82.24	0.9134
		Maximum	86.13	86.81	86.13	86.04	0.9377
20 MrMr (4 bands)	Top 20	Average	78.35	78.75	78.35	78.24	0.8704
		Maximum	82.18	82.18	82.18	82.18	0.9049
10 MrMr (4 bands)	Top 10	Average	60.45	60.99	60.45	59.62	0.6324
		Maximum	67.24	68.12	67.24	66.67	0.7245

ReliefF performed stronger than MrMr across the experiments, with the Top 30 ReliefF-selected features yielding maximum accuracy of 98.26% for (Advanced SNN) and 97.68% for (Proposed DNN). These findings highlight the importance of a sufficiently rich feature set capturing important discriminative data patterns needed for more reliable classification. On the contrary, Top 30 MrMr-selected features show a clear reduction in performance. Under this setting, the Proposed DNN achieved a maximum accuracy of 86.13%.

The proposed DNN performs better than the Advanced SNN across most feature settings. This reflects the ability of the model to learn complex relationships in EEG-derived features. The delta and theta bands repeatedly give the strongest results which further support their importance in EEG-based dyslexia classification. Meanwhile, when full combined feature set was used, performance drops slightly. A most possible reason for this tis that the fused set also includes weaker or less relevant descriptors. Those extra features can add noise and reduce how well the model generalizes.

In conclusion, the most consistent performance is reported with larger feature sizes for the ReliefF-based subsets, while subsets from MrMr present a decrease in performance with this dataset. Delta and theta are the most discriminative of the frequency bands for this classification task. In this instance, the proposed DNN surpasses the Advanced SNN in all feature sets.

#### 3.3.1 Justification

The superior performance results reported for ReliefF-based feature subsets, particularly with larger feature size, demonstrates the effectiveness of the ReliefF algorithm in identifying and ranking the most informative features for dyslexia classification. The decline in performance is observed when using MrMr-selected features with smaller subset size. This suggests that even though MrMr is effective at minimizing redundancy, the aggressive removal of correlated features can also eliminate variables that carry useful class-specific information. As a result, key discriminative features may be lost, leading to drop in classification performance. The consistently strong results obtained for delta and theta features support their importance in EEG-based classification tasks, as these frequency bands report neural activity associated with dyslexia. As for the lower performance of combined features is concerned, it is attributed to including less informative features. These introduce noise and reduce the model’s generalization ability. Overall, the deep network delivers more consistent and higher performance than the advanced shallow network, indicating that the proposed DNN is better able to learn complex, non-linear patterns from EEG-derived features.

### 3.4 Analysis of top-performing models across band-specific and feature selection approach

This section covers the performance evaluation of top-performed models across both band-wise features as well as feature selection approaches. The classification experiments presented in Table 8 confirm that ensemble-based approaches produce best results compared to traditional classifiers across both individual and combined features. ESD achieved the highest accuracy (86.93%) for the alpha band, closely followed by Ensemble Bagged Trees (85.83%) and Ensemble Boosted Trees with RUS (86.18%). On the other hand, simpler classifiers, such as Medium Tree (76.97%) and Coarse Tree (80.54%) provided competitive baseline performance.

In the beta band, EBT-RUS and ESD demonstrated effectiveness of ensemble methods in capturing complex patterns by achieving the highest accuracies of 88.25 and 88.08%, respectively. Among simpler models, Medium Tree highlights its reliability by achieving an accuracy of 80.37%. Similarly, ensemble methods such as Bagged Trees and Boosted Trees with RUS consistently achieved accuracies above 85% in the delta and theta bands. These approaches surpass traditional classifiers such as Medium Tree by scoring 78.29 and 80.42% for Delta and Theta bands, respectively.

When using the combined feature set, Ensemble Boosted Trees achieved the highest accuracy (96.43%), followed by Ensemble Bagged Trees (96.03%) and Ensemble Subspace Discriminant (96.20%). However, Tree-based models such as Medium Tree demonstrate competitive results (94.13%) but were outperformed by advanced ensemble-based approaches. Overall, ensemble-based models demonstrate excellent performance with the combined set yielding best results by utilizing complex patterns within individual bands as well as combined features. The findings summarized above confirm the robustness of ensemble-based methods for accurate classification of dyslexia using EEG signals.

The best classification results with key insights into the performance of various classifiers and feature sets for dyslexia detection are presented in Table 8. Among the individual EEG bands, Beta features demonstrated the best overall performance with an accuracy of 88.25%, a specificity of 90.62%. However, Ensemble Boosted Trees with RUS demonstrate the highest AUC of 0.9457, indicating that Beta band features are particularly discriminative for the task. The Alpha band also showed strong performance using Ensemble Subspace Discriminant with an accuracy of 86.93% and an AUC of 0.9385, which further supports the utility of individual bands for classification. In addition, Delta and Theta bands demonstrated slightly lower accuracies (84.97 and 85.89%, respectively) but showed reliable classification potential, particularly with Ensemble Bagged Trees. The combination of all EEG bands significantly improved the performance of models. The best results, accuracy (96.43%), sensitivity (96.51%), specificity (96.50%), and an AUC (0.9643) were achieved in Ensemble Boosted Trees. The combined features likely capture complementary patterns across the EEG spectrum thereby underscoring the importance of leveraging comprehensive feature information to enhance classification.

ReliefF consistently outperformed MrMr for feature selection methods. LDA outperformed all with accuracy (85.02%), sensitivity (85.71%), and a high AUC (0.9374) by using the Top 30 ReliefF-selected features. On contrary, MrMr’s Top 30 features yielded a lower accuracy of 73.51% and an AUC of 0.8049 by utilizing Ensemble Boosted Trees with RUS. These findings suggest ReliefF-selected features are more informative and best suited for classification-based tasks, in particular with simpler classifiers like LDA.

Both MrMr and ReliefF feature selection techniques demonstrate better results without feature selection. However, their underlying mechanisms differ significantly thereby showing the slight variation of results. This is attributed to MrMr feature selection which is entirely based on mutual information gain, optimizing relevance to target and redundancy among features. This approach is well-suited for identification of linearly dependent features but weaker to capture complex feature interactions. On the contrary, the ReliefF algorithm is entirely based on feature importance analysis between the features that better distinguish between neighboring instances of two different classes. This allows for capturing complex non-linear relationships and local interactions among the features, thereby capturing context-specific subtle patterns in the EEG data. Therefore, in the domain of EEG-based dyslexia detection, where non-linear and highly complex interactions dominate. As a result, ReliefF consistently outperforms MrMr in the domain of EEG-based dyslexia detection which are dominated by non-linear and highly complex interactions.

The proposed Deep Neural Networks (DNN) achieved the highest overall performance by utilizing the Top 20 ReliefF features, with an exceptional accuracy (98.85%), sensitivity (98.88%), specificity (98.88%), and an AUC of 0.9984 as shown in Table 9. These findings highlight the power of deep learning to exploit compact, highly informative feature sets, extracting complex patterns that are otherwise ignored by traditional methods.

**Table 9:** Top best performing models across band-specific features and feature selection approach.

Input	Classifier	Accuracy (%)	Sensitivity (%)	Specificity (%)	Auc
Alpha	ESD	86.93	86.91	87.54	0.9385
Beta	EBT- RUS	88.25	87.01	90.62	0.9457
Delta	Ensemble bagged trees	84.97	84.35	86.91	0.9156
Theta	Ensemble bagged trees	85.89	85.21	87.69	0.8589
All combined	Ensemble boosted trees	96.43	96.51	96.50	0.9643
MrMr (Top 30)	EBT-RUS	73.51	74.49	73.46	0.8049
ReliefF (Top 30)	LDA	85.02	85.71	84.44	0.9374
ReliefF (Top 20)	Proposed deep neural network	98.85	98.88	98.85	0.9984

Although the traditional ML classifiers required very little computational time, as shown in Table 10, their overall performance remained comparatively limited across the evaluation metrics. In contrast, the proposed DNN achieved stronger results while still maintaining a practical runtime of 11.3 s. The model’s deep architectural framework helps it capture complex and non-linear relationships within the EEG signal well. Thus, higher training time is a worthwhile trade-off, especially in real-world clinical applications where inference is prioritized over training speed, which has higher priority over training time. The proposed Deep NN is the most effective model for this task.

**Table 10:** Training time of models.

Model	Training time (seconds)	Model	Training time (seconds)
Fine tree	0.0029	Fine KNN (*k* = 3)	0.0022
Medium tree	0.0043	Medium KNN (*k* = 10)	0.0014
Coarse tree	0.0059	Coarse KNN (*k* = 100)	0.0015
LDA	0.0013	Weighted KNN (*k* = 20)	0.0013
QDA	0.0009	Cosine KNN (*k* = 10)	0.0069
LR	0.0030	Cubic KNN (*k* = 10)	0.0020
GNB	0.0008	Ensemble boosted trees	0.3029
Linear SVM	0.8031	Ensemble bagged trees	0.743
Quadratic SVM	0.2253	ESD	0.469
Cubic SVM	0.3281	EBT- RUS	0.502
Fine Gaussian SVM	0.3108	Shallow NN	8.005
Medium Gaussian SVM	0.3825	Deep NN	11.338
Coarse Gaussian SVM	0.3533		

**Figure 4:**
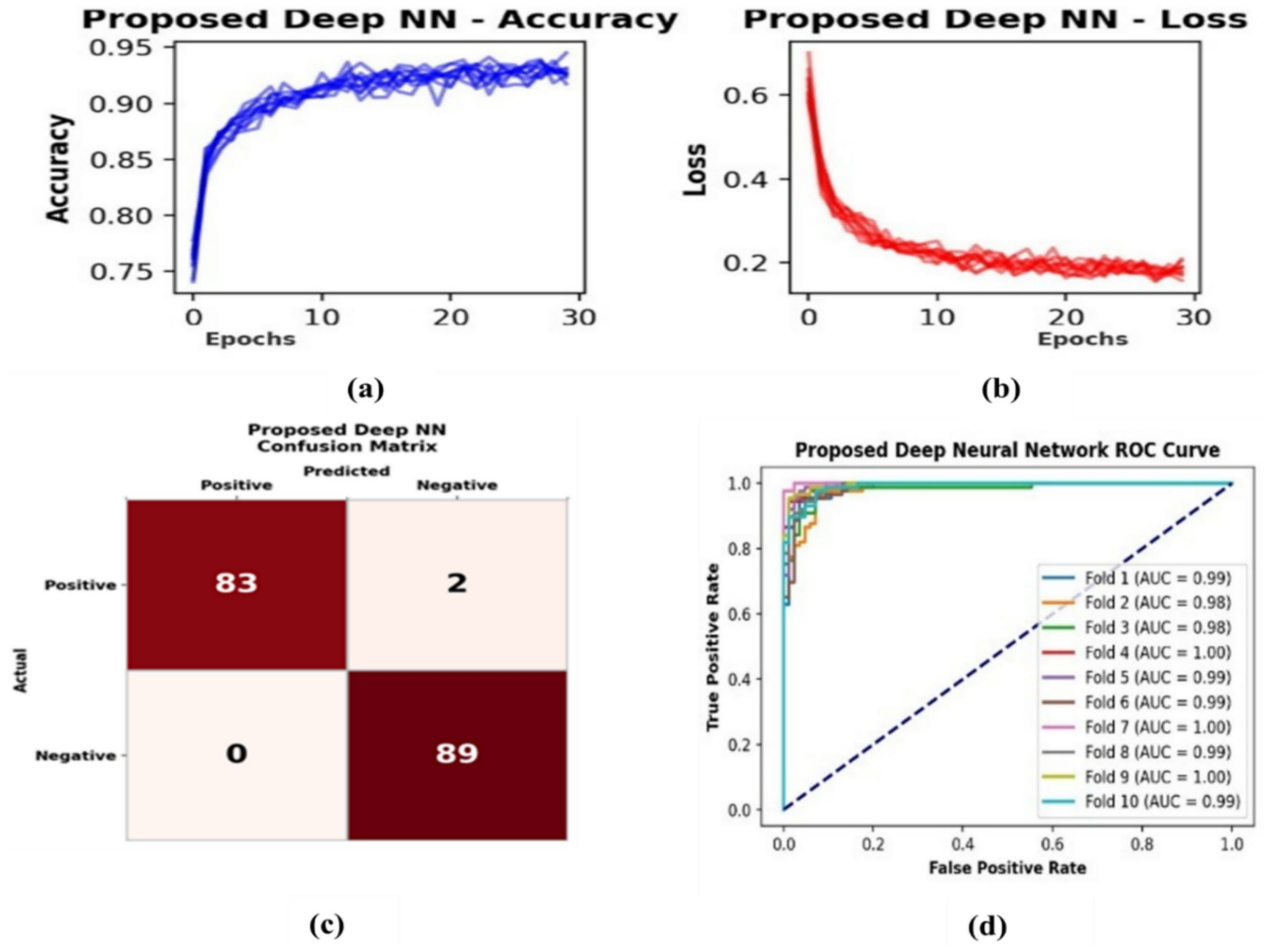
Performance results of the proposed deep NN. **(a)** Training accuracy across epochs shows rapid convergence, stabilizing around ~0.93–0.94 after the early epochs. **(b)** Training loss decreases steadily from ~0.65 to ~0.18, demonstrating improved model fit wit with no obvious instability. **(c)** Confusion matrix from one fold of the test set shows near-perfect classification (TP = 83, TN = 89, FP = 0, FN = 2), corresponding to ~98.9% accuracy, ~97.6% sensitivity, and 100% specificity. **(d)** ROC curves from 10-fold cross-validation lie near the top-left corner with AUC ≈ 0.98–1.00, confirming excellent discriminative ability and very low false positive rates across folds.

Figure 4 above presents the performance results of the proposed deep neural network for EEG-based dyslexia detection. The subplot illustrates the training accuracy and loss across multiple epochs, showing a consistent, smooth trend indicating a stable learning behavior. This steady decrease in loss confirms effective optimization and limited overfitting. The consistent convergence of loss curves and stable training accuracy across folds affirms that the proposed DNN did not overfit despite its limited size, with stratified 10-fold cross-validation providing robust internal validation of the model. However, because evalaution is carried out at the segment level (multiple epochs per child), Subject-level overlap (segments from same participant may appear in both training and test folds) between folds may occur, which can inflate performance results due to intra-subject correlation. Therefore, future work will further implement subject-wise cross-validation and external dataset validation to enhance generalizability and robustness of the proposed model. The third subplot shows the confusion matrix of the one-fold on the test set, where the proposed model correctly classifies 83 instances out of 85 non-dyslexic samples and 89 out of 89 dyslexic samples, reflecting a high classification accuracy with minimal misclassifications. Finally, the ROC curve for each fold in the 10-fold cross-validation (as shown in the fourth subplot) reveals consistently high AUC values, demonstrating the model’s excellent ability to discriminate between the two classes across all folds. These results correctly confirm the proposed model’s robustness and high predictive performance.

#### 3.4.1 Key justifications

- *Beta Band Dominance*: The beta band’s high AUC and specificity values highlight its strong discriminative power/capability, due to cognitive and attention-related activities associated with dyslexia.
- *Combined Features Advantage*: The significant improvement in combined features validates the hypothesis that diverse feature information enhances model performance by capturing broader EEG dynamics.
- *ReliefF Superiority*: ReliefF’s consistent performance indicates its ability to manage features with maximum inter-class separability, especially with the LDA model.
- *Deep Learning Excellence*: The strong performance results of the proposed DNN shows its capability to utilize the high-level abstractions of compact feature set, which is a characteristic of complex noisy EEG datasets.

### 3.5 Comparison with existing studies on our dataset

To examine cross-dataset generalization, we have evaluated and re-implemented representative existing pipelines on our dataset using the same preprocessing and results are reported under respective evaluation protocol of each method. Table 11 describes the originally reported results of the pipelines and the corresponding performance obtained on our own dataset. Experimental results reported in Table 11 show transferability of each approach. The feature-based ML approaches showed moderate-to-strong transferability, while other specialized pipelines exhibited reduced portability. In particular, Perera et al. (2018) and Parmar and Paunwala (2023a) achieved accuracies of 86.33 and 86.66%, respectively, on our dataset, and Christodoulides et al. (2022) obtained accuracy of 90.33% (Sens = 94.16%, Spec = 90.83%). The ensemble-voting approach of Zaree et al. (2023) obtained 88.39% (Sens = 85.41%, Spec = 92.70%, F1 = 87.79%). In contrast, the graph-based method of Gallego-Molina et al. (2022) achieved 60.90% on our dataset, indicating sensitivity to dataset/task differences. It is important to mention that, methods such as Guhan Seshadri et al. (2023) and Parmar and Paunwala (2023b) reported very high accuracies 82.33 and 75.86% respectively, when evaluated under same validation protocol as done for our proposed approach. The results documented in Table 11, highlight the importance of consistent evaluation when assessing generalization across datasets.

**Table 11:** Comparison of proposed DNN with existing state-of-art EEG methods.

References	Data (electrodes)	Dataset size (Dys/N-Dys)	Approach	Features	Classifiers	Results reported	Results on our dataset
Perera et al. (2018)	EEG (32)	17/15	Machine learning	Statistical and frequency sub-band decomposition	Cubic SVM	Acc = 78.2%	Acc = 86.33% Sens = 87.49% Spec = 90.83% F1-score = 86.57%
Christodoulides et al. (2022)	EEG (12)	12/14	Machine learning	Filtering energy in bands across different brain regions	SVM, DT, KNN, NB, RF Best = RF	Acc = 87.04% Sens = 90.91% Spec = 80.95%	Acc = 90.33% Sens = 94.16% Spec = 90.83%
Gallego-Molina et al. (2022)	EEG (19)	16/32	Graph Analysis	Clustering coefficient, Small worldness, path length, assortativity, degree, density	Support Vector Machine	Acc = 72.9% Pre = 72.3% Rec = 74.9%	Acc = 60.90% Sens = 88% Spec = 32%
Zaree et al. (2023)	EEG (19)	22/22	Ensemble Voting	14 (amplitude and latency of 7 components)	SVM, DT, LDA, KNN, and Naive Bayesian	Acc = 87.5% Sens = 81.2% Spec = 93.7%	Acc = 88.39% Sens = 85.41% Spec = 92.70% F1-score = 87.79%
Parmar and Paunwala (2023a)	EEG (19)	24/29	Machine Learning	Power spectrums, Slow wave index, Average band power, Hjorth mobility and Complexity	SVM (Poly Kernel) and ReliefF Algorithm	Acc = 79.3%	Acc = 86.66% Pre = 90.83% Rec = 90.00% F1-Score = 89.90%
Guhan Seshadri et al. (2023)	EEG (19)	20/20	Neural Networks	Statistical Features like mean, median, mode, kurtosis,	Shallow Neural Network	Acc = 97.5% Sens = 100% Spec = 95%	Acc = 82.33% Pre = 85.00% Rec = 70.83% F1-Score = 76.23%
Parmar and Paunwala (2023b)	EEG (19)	15/15 15 healthy used for evaluation	Machine Learning	Wavelet Scattering Transform	SVM (RBF Kernel)	Acc = 98.67%	Acc = 75.86% Pre = 81.49% Rec = 75.86%
Proposed work	EEG (19)	26/25	Deep learning	Statistical Features and DWT subjected to extraction using MrMr and ReliefF feature selection method	Deep Neural Network	Acc = 98.85% Sens = 98.88% Spec = 98.85%	

The results suggest that selecting effective feature selection technique, combined with advanced classification models, is critical for achieving reliable dyslexia detection outcomes. As summarized in Table 10, the proposed approach outperformed all existing approaches that have attempted to classify dyslexia using EEG-based approaches combined with machine learning. Most of the prior works used a limited number of electrodes, resting state EEG. They relied on single-feature selection techniques, such as ReliefF (Parmar and Paunwala, 2023a) and a graph-based approach (Guhan Seshadri et al., 2023), resulting in moderate performance.

In contrast, the present study advances EEG-based dyslexia detection through a dual feature selection strategy and deep learning to achieve superior model performance. Features derived from statistical descriptors and wavelet-domain coefficients capture temporal-spectral patterns, while the combined use of MrMr and ReliefF balances feature relevance and redundancy enhancing discriminative power and generalization (Parmar and Paunwala, 2023b). Using multi-resolution wavelet decomposition together with dual feature selection (ReliefF and MrMr), and a deep neural network optimized for generalization, our proposed approach achieves higher accuracy of 98.85%, with perfect balance across accuracy, precision, recall, F1-Score, and AUC, outperforming SNN of Guhan Seshadri et al. (2023) with accuracy of 97.5%. This enhanced performance is supported by four hidden layers instead of three (SSN), allowing the model to learn more complex patterns and non-linear relationships without overfitting. Additionally, the inclusion of batch normalization and dropout layers at every stage enhanced the regularization and training stability for reliable classification performance.

In comparison to Ortiz et al. (2020), who employed SSA-CNN using the component correlation matrices derived from Singular Spectrum Analysis, our proposed approach simplifies the preprocessing pipeline via db4 wavelets and ICA, as detailed in Section 2.5. This design is computationally more efficient and better suited for real time portable screening. These enhancements in model design not only increases accuracy but also improved its real-time deployment feasibility. This led to strong diagnostic performance, achieving sensitivity of (98.88%) and a specificity of (98.85%).

In order to further understand the individual contribution of EEG features, the ReliefF and MrMr algorithms were used to evaluate the most important discriminative frequency bands and EEG channels. The delta and theta bands emerged as dominant contributors, supporting previously reported findings about presence of slow wave oscillations in the regions implicated in dyslexia. Spatially, the frontal-temporal (F3, F4, T3, T4) and parietal (P3, P4) electrodes exhibited greater activity which reflects the role of phonological processing, attention, and working memory in these tasks. These findings indicate that the model’s performance is primarily driven by neurocognitively meaningful features, aligning with existing neurocognitive accounts of dyslexia and strengthening the interpretation of the results. However, these EEG signatures should not be interpreted as uniquely dyslexia-specific biomarkers, because similar spectral and task-evoked EEG alterations may arise from broader neurodevelopmental or cognitive factors such as executive function and language processing, that can overlap with reading difficulties. In this study, participants were clinically evaluated and the dataset was restricted to children (excluding diagnosed comorbid neurodevelopmental disorders and global cognitive impairment), which reduces potential confounding. Nevertheless, the present design does not include dedicated clinical comparison groups (ADHD/ASD/developmental language disorder) to quantify disorder specificity. Therefore, the proposed framework should be interpreted as a screening classifier aligned with DALI-based labeling, and future work will evaluate multi-group cohorts to establish differential specificity.

A key strength of this study is the use of a larger and well-balanced cohort of 51 participants (26 dyslexic and 25 non-dyslexic) compared with the smaller samples commonly reported in past studies (Perera et al., 2018; Christodoulides et al., 2022; Zaree et al., 2023). This more balanced design reduces class bias and supports stronger generalization across individuals.

The novelty of the proposed approach lies in the methodological pipeline based on DWT-driven feature engineering and the contextual originality of the dataset. Unlike previous studies that usually rely on public datasets or resting state EEG, our study utilizes task-evoked EEG data collected from children during learning related activities in real educational settings. This makes the signals more closely tied to dyslexia-relevant cognitive functions, improves ecological validity, and increases the practical relevance of the framework for early screening in local populations. While the proposed DNN model employed is intentionally compact and standard, the performance gains are primarily driven by the wavelet-domain representation and feature-selection pipeline. Collectively, these aspects represent an important step toward building population-specific, deployable screening tools for early dyslexia detection.

## 4 Ablation study

### 4.1 Analysis on four-band vs. five-band features

To understand the impact of feature representation and frequency-band inclusion, we performed an ablation comparing the proposed DNN under two settings: (i) four-band features (*δ*, *θ*, *α*, *β*) and (ii) five-band features (δ, θ, α, β, *γ*). For the four-band pipeline, we evaluated both single-band features (160 features per band) and the combined four-band feature vector (640 features), as well as feature-selection variants (ReliefF and MrMr). The four-band results show that combining bands improves robustness: the combined 4-band feature vector achieved 93.08% average accuracy (AUC 0.9834), outperforming individual bands (Delta: 92.79%, Theta: 89.23%, Alpha: 85.90%, Beta: 80.14%). Further, ReliefF-based selection consistently improved performance, with ReliefF Top-20 achieving the best overall results (95.27% average accuracy and AUC 0.9930, with a maximum accuracy of 98.85%), whereas MrMr selections reduced performance substantially (MrMr Top-30 average 82.27%, Top-10 average 60.45%), indicating inferior feature ranking for this dataset.

In the five-band ablation, we extended the feature extraction by including the *γ* band and repeated the same evaluation using full features and Top-k selection and results are summarized in Table 12. The combined 5-band full feature set achieved 87.95% mean accuracy (F1 88.72%, AUC 0.9525), and MrMr Top-30 produced a marginal improvement (88.17% mean accuracy, F1 89.01%, AUC 0.9517). In contrast, ReliefF-based selection in the five-band setting resulted in noticeably lower performance (mean accuracy 70.59–79.19%, AUC 0.7718–0.9093), suggesting that adding γ introduces features that are more sensitive to high-frequency noise/EMG contamination and can degrade stability depending on the selector. To directly assess the contribution of gamma band features, we also evaluated Gamma-only features, which achieved 81.18% accuracy, 80.34% precision, 91.26% recall, 85.45% F1-score, and 0.9087 AUC. However, compared with the four-band results in Table 8, the five-band setting does not provide a consistent improvement, and its performance depends strongly on the feature-selection method (MrMr remains stable whereas ReliefF degrades notably). Overall, the ablation study indicates that the four-band configuration is more robust and consistently high-performing for our dataset, while the five-band (γ-included) setting does not provide a consistent advantage and may increase variability across selection methods. Therefore, we retain the four-band setting as the primary configuration and report five-band results as an ablation for completeness.

**Table 12:** Five-band (δ, θ, α, β, γ) ablation results for the proposed DNN.

Feature set	Input size	Accuracy (%)	Precision (%)	Recall (%)	F1 score (%)	Roc
Gamma band	160	81.1765	80.3419	91.2621	85.4545	0.908709
Combined features (5 bands)	800	87.9550	86.4908	92.4320	88.7218	0.952511
MrMr Top 10 (combined 5 band)	10	86.1315	84.6431	91.1964	87.2520	0.941824
MrMr Top 20 (combined 5 band)	20	87.0750	85.6421	91.6918	87.8891	0.948169
MrMr Top 30 (combined 5 band)	30	88.1705	86.6811	91.9288	89.0058	0.951677
ReliefF Top 10 (combined 5 band)	10	70.5882	74.7664	77.6699	76.1905	0.771772
ReliefF Top 20 (combined 5 band)	20	72.9412	78.2178	76.6990	77.4510	0.835386
ReliefF Top 30 (combined 5 band)	30	79.1908	99.0566	75.0000	85.3659	0.909307

### 4.2 Analysis on using raw EEG deep learning baselines

To examine whether the performance of the proposed DNN depends on handcrafted DWT features or can be achieved directly from time-domain EEG, we performed an ablation using end-to-end deep learning models trained on raw EEG epochs (10-s epochs; 2,560 samples at 256 Hz × 16 channels, 1,734 epochs from 51 subjects). The raw baselines were evaluated using the same protocol and are summarized in Table 13. The raw-signal models achieved moderate performance: EEGNet performed best with mean Accuracy = 88.26%, F1 = 86.76%, and AUC = 0.9549, followed by LSTM (mean Accuracy = 82.77%, F1 = 82.53%, AUC = 0.9474) and 1D-CNN (mean Accuracy = 79.12%, F1 = 75.76%, AUC = 0.9582). Compared with the proposed DWT feature-based DNN (Table 8) (≈93–96% mean accuracy and 98.85% maximum), the raw-EEG baselines show lower accuracy/F1 and greater variability, indicating that the DWT-based representation provides a more compact and sample-efficient feature space for dyslexia screening in our dataset and recording setting, while raw end-to-end learning remains a viable but currently less effective alternative.

**Table 13:** Ablation study—end-to-end learning from raw EEG compared to the proposed feature-based DNN pipeline.

Model	Input (per epoch)	Accuracy (%)	Precision (%)	Recall (%)	F1-Score (%)	Auc
1D-CNN	2,560 × 16	79.12	88.99	79.11	75.76	0.9582
EEGNet	2,560 × 16 (reshaped to 16 × 2,560 × 1)	88.26	90.20	87.19	86.76	0.9549
LSTM	2,560 × 16	82.77	90.77	80.43	82.53	0.9474

### 4.3 Wavelet-domain statistical characterization

**Figure 5:**
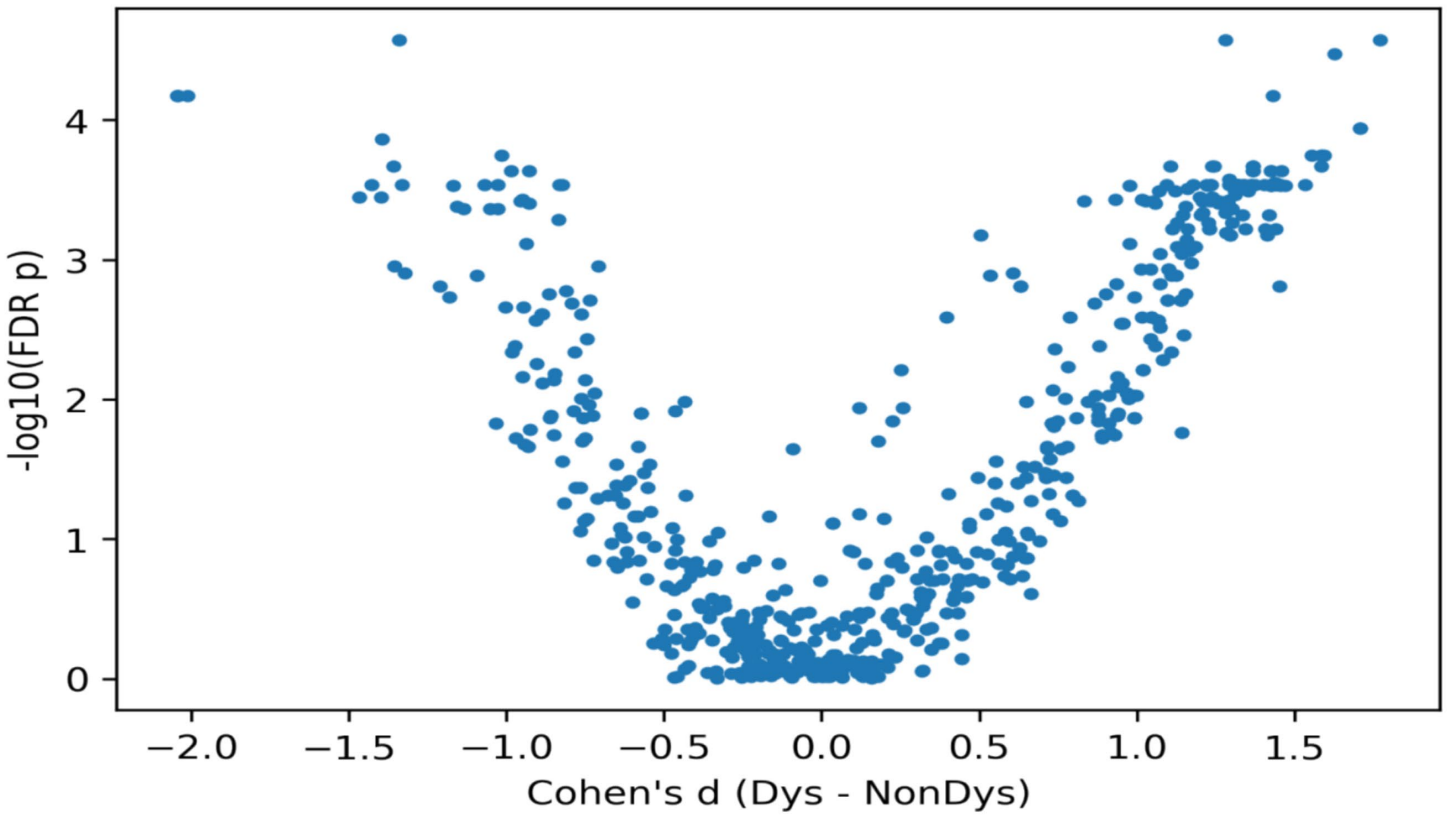
Wavelet-domain feature statistics. Scatter plot of effect size (Cohen’s *d*, dyslexic–non-dyslexic) versus −log_10_(FDR-adjusted *p*-value) for wavelet-derived features. Higher *y*-values indicate stronger statistical significance after multiple-comparison correction; larger |*d*| indicates stronger class separation.

To support the use of DWT with signal-level evidence, we analyzed class-wise differences in the distributions of wavelet-domain features (derived from DWT coefficients) using statistical testing with FDR correction. A total of 319 features were significant after FDR correction, indicating consistent group differences in wavelet-domain activity. Overall, 319/640 (49.8%) features remained significant after FDR correction. The band-wise distribution of significant features was highest in beta (109/160; 68.1%), followed by delta (80/160; 50.0%) and alpha (77/160; 48.1%), with theta showing fewer significant features (53/160; 33.1%). The strongest effects were observed in the beta and delta band, particularly over frontal channels (such as F3), where multiple descriptors (mean/median, variance/STD, RMS, entropy, IQR, MAD) showed very low *p*-values (≈10^−7^–10^−6^) and large effect sizes (|d| > 1). Significant differences were also present in theta and alpha features across several channels, supporting that discriminative information exists at the signal-statistics level in addition to classification performance. Figure 5 shows Cohen’s d versus −log10(FDR-adjusted p) for all wavelet-derived features. A large number of features exhibit both large effect sizes and significant FDR-corrected p-values, confirming that discriminative information exists at the signal/feature distribution level. Overall, 319 features remained significant after FDR correction, supporting the relevance of DWT-based representations in our dataset.

## 5 Limitations and future scope

Despite promising results, the research study has certain limitations. First, although the sample size is larger than most dyslexia studies, it was pre-specified using a nomogram-guided feasibility approach to achieve a minimum informative diagnostic sample. However, it is still relatively small and remains restricted to the 5–10-year age range. At the same time, psychologists have intentionally selected this range to target the early childhood, a critical and more effective period for dyslexia screening and intervention. We acknowledge that this limited sample size and age-specific focus has limited the generalizability of proposed DNN to varying age groups with different neural and compensatory mechanisms. To overcome this limitation, future studies aim to increase the dataset size and learning deficit types across broader age ranges to include demographic and clinical generalizability.

Second, the proposed model evalaution was conducted using segment-level stratified 10-fold cross-validation, where multiple EEG epochs from same child may be distributed across different folds. This subject -level overlap can lead to inflated performance estimates. Therefore, future work will implement subject-level cross-validation and external cohort validation to assess generalizability on unseen children.

Third, we did not report quantitative EEG quality metrics (e.g., impedance/SNR). Although filtering and ICA reduced artifacts, some task-related contamination may remain and signal quality can vary across sessions. Future work will incorporate standardized automated IC labeling and additional quality-control metrics to further improve robustness in real-world deployments.

Fourth, dyslexia is heterogeneous and can vary in severity; however, in this work we framed the task as a binary screening problem aligned with the DALI tool categorization (dyslexic vs. non-dyslexic). This formulation does not capture severity gradients or subtype variation. Future work will extend the framework to severity-aware or multi-class modeling when larger datasets with graded clinical annotations are available. Additionally, because the study did not include clinically characterized comparison groups (such as ADHD, ASD, developmental language disorder), the dyslexia-specificity of the identified EEG signatures cannot yet be established and requires multi-condition validation in future work.

Furthermore, future work will incorporate advanced feature selection techniques like embedded feature selection, mutual information, or PCA, and more advanced deep learning architectures like CNN and attention mechanisms to improve further performance and generalizability. Future research will also explore multimodal approaches that combine EEG with behavioral and eye-tracking data for effectively capturing neural and behavioral biomarkers linked to dyslexia, thereby enhancing diagnostic precision and model interpretability.

Finally, the proposed framework can be translated into a fully portable screening tool for clinicians and educators by integrating wireless, portable EEG headsets. Practical translation would require streamlined electrode setup, automated signal quality checks and artifact handling and fast inference so that results can be generated within minutes. We will evaluate robustness under real-world conditions(device variability, motion artifacts and classroom noise), and assess usability factors such as setup time, screening accuracy, and operator training to support scalable, low-cost screening. With this setting, the proposed framework aims to enable affordable, onsite, and easy to operate screening in schools and clinical environments for more accurate screening outcomes and targeted intervention.

## 6 Conclusion

The study presents a practical framework for classifying dyslexic and non-dyslexic children (5–10 years) using EEG data and advanced machine learning techniques. The workflow includes task-based EEG acquisition from participants, followed by systematic signal processing, segmentation and decomposition into *α*, *β*, *δ*, and *θ* bands, enabling handcrafted feature extraction from highly non-stationary EEG signals. These dominant features were then subjected to feature selection using a dual feature selection strategy using the RelieF and MrMr algorithms, focusing on using a varying number of features. The top-ranked feature subsets were used to train models with the proposed DNN, achieving the highest maximum accuracy of 98.85%, outperforming existing EEG-based dyslexia detection systems, as summarized in Table 11. These research findings highlight the effectiveness of combining DWT for multi-resolution signal processing with dual feature selection for robust feature optimization, and deep learning for high precision classification. In addition, we conducted systematic ablation studies (band-wise, fused bands, and Top-k feature subsets) and re-implemented representative existing pipelines and raw-EEG deep learning baselines (1D-CNN, EEGNet, and LSTM) on our dataset under the same validation protocol, providing a fair benchmarking of generalization and reinforcing the robustness of the proposed framework.

## Data Availability

The dataset used in this study is not publicly available due to privacy and ethical considerations but can be accessed upon reasonable request. It will be made available exclusively for research purposes by Tabassum Gull Jan.

## References

[ref1] Al-Barhamtoshy H. M. Motaweh D. M., “Diagnosis of dyslexia using computation analysis,” 2017 International Conference on Informatics, Health and Technology (ICIHT 2017), Riyadh Saudi Arabia: IEEE. (2017).

[ref2] Aljalal M. Aldosari S. A. Molinas M. AlSharabi K. Alturki F. A. (2022). Detection of Parkinson’s disease from EEG signals using discrete wavelet transform, different entropy measures, and machine learning techniques. Sci. Rep. 12, 1–19. doi: 10.1038/s41598-022-26644-7, 36581646 PMC9800369

[ref3] Alkhrijah Y. Khalid S. Usman S. M. Jameel A. Zubair M. Aldossary H. . (2025). Feature fusion ensemble classification approach for epileptic seizure prediction using electroencephalographic bio-signals. Front. Med. 12:1566870. doi: 10.3389/fmed.2025.1566870, 40832093 PMC12358471

[ref4] Alkhurayyif Y. Sait A. R. W. (2024). Multi-modal dyslexia detection model via SWIN transformer with closed-form continuous time networks. IEEE Access 12, 127580–127591. doi: 10.1109/ACCESS.2024.3454795

[ref5] Başar E. Başar-Eroglu C. Karakaş S. Schürmann M. (2001). Gamma, alpha, delta, and theta oscillations govern cognitive processes. Int. J. Psychophysiol. 39, 241–248. doi: 10.1016/S0167-8760(00)00145-8, 11163901

[ref6] Bejani M. M. Ghatee M. (2021). A systematic review on overfitting control in shallow and deep neural networks. Artif. Intell. Rev. 54, 6391–6438. doi: 10.1007/s10462-021-09975-1

[ref7] Chaabene S. Bouaziz B. Boudaya A. Hökelmann A. Ammar A. Chaari L. (2021). Convolutional neural network for drowsiness detection using eeg signals. Sensors 21, 1–19. doi: 10.3390/s21051734, 33802357 PMC7959292

[ref8] Che J. Yang Y. Li L. Bai X. Zhang S. Deng C. (2017). Maximum relevance minimum common redundancy feature selection for nonlinear data. Inf. Sci. 409–410, 68–86. doi: 10.1016/j.ins.2017.05.013

[ref9] Christodoulides P. Miltiadous A. Tzimourta K. D. Peschos D. Ntritsos G. Zakopoulou V. . (2022). Classification of EEG signals from young adults with dyslexia combining a brain computer Interface device and an interactive linguistic software tool. Biomed. Signal Process. Control 76:103646. doi: 10.1016/j.bspc.2022.103646

[ref10] Delorme A. Makeig S. (2004). EEGLAB: an open source toolbox for analysis of single-trial EEG dynamics including independent component analysis. J. Neurosci. Methods 134, 9–21. doi: 10.1016/j.jneumeth.2003.10.009, 15102499

[ref11] Demir A. Koike-Akino T. Wang Y. Haruna M. Erdogmus D. “EEG-GNN: graph neural networks for classification of electroencephalogram (EEG) signals,” Annual International Conference of the IEEE Engineering in Medicine and Biology Society. 1061–1067. Mexico: IEEE. (2021). 10.1109/EMBC46164.2021.9630194 34891471

[ref12] Edderbali F. Harmouchi M. Essoukaki E. (2023). “Classification of EEG signal based on pre-trained 2D CNN model for epilepsy detection,” in Digital Technologies and Applications, Switzerland: Cham and Springer Nature. 1008–1016.

[ref13] Fajariyanti F. M. Agata D. Harsono T. (2022). Expert system for learning disability classification in school-age children. Proc. Int. Conf. Appl. Sci. Technol. Soc. Sci 647, 231–237. doi: 10.2991/assehr.k.220301.039

[ref14] Formoso M. A. Ortiz A. Martínez-Murcia F. J. Brítez D. A. Escobar J. J. Luque J. L. (2022). Temporal phase synchrony disruption in dyslexia: anomaly patterns in auditory processing. Lect. Notes Comput. Sci 13258, 13–22. doi: 10.1007/978-3-031-06242-1_2

[ref15] Fries P. (2005). A mechanism for cognitive dynamics: neuronal communication through neuronal coherence. Trends Cogn. Sci. 9, 474–480. doi: 10.1016/j.tics.2005.08.011, 16150631

[ref16] Gallego-Molina N. J. Ortiz A. Martínez-Murcia F. J. Formoso M. A. Giménez A. (2022). Complex network modeling of EEG band coupling in dyslexia: an exploratory analysis of auditory processing and diagnosis. Knowl. Based Syst. 240:108098. doi: 10.1016/j.knosys.2021.108098

[ref17] Gallego-Molina N. J. Ortiz A. Martínez-Murcia F. J. Rodríguez-Rodríguez I. (2022). Unraveling dyslexia-related connectivity patterns in EEG signals by Holo-Hilbert spectral analysis. Lect. Notes Comput. Sci 13258, 43–52. doi: 10.1007/978-3-031-06242-1_5

[ref18] Guhan Seshadri N. P. Agrawal S. Kumar Singh B. Geethanjali B. Mahesh V. Pachori R. B. (2023). EEG based classification of children with learning disabilities using shallow and deep neural network. Biomed. Signal Process. Control 82:104553. doi: 10.1016/j.bspc.2022.104553

[ref19] Guhan Seshadri N. P. Singh B. K. Pachori R. B. (2023). EEG based functional brain network analysis and classification of dyslexic children during sustained attention task. IEEE Trans. Neural Syst. Rehabil. Eng. 31, 4672–4682. doi: 10.1109/TNSRE.2023.3335806, 37988207

[ref20] Hanafi M. F. M. Mansor W. Zainuddin A. Z. A. (2023). Recognition of EEG signals of dyslexic children using long short-term memory. AIP Conf. Proc 2562:060002-1–060002-8. doi: 10.1063/5.0112606

[ref21] Hyvärinen A. Oja E. (2000). Independent component analysis: algorithms and applications. Neural Netw. 13, 411–430. doi: 10.1016/S0893-6080(00)00026-5 10946390

[ref22] Ileri R. Latifoglu F. Demirci E. (2020). “New method to diagnosis of dyslexia using 1D-CNN,” in TIPTEKNO 2020 - Tıp Teknolojileri Kongresi- 2020 Medical Technologies Congress (TIPTEKNO) TIPTEKNO 2020, (5–8.

[ref23] Jan T. G. Khan S. M. (2023). An Effective Feature Selection and Classification Technique Based on Ensemble Learning for Dyslexia Detection BT - Intelligent Communication Technologies and Virtual Mobile Networks, Singapore: Springer Nature Singapore. 413–423.

[ref24] Jothi Prabha A. Bhargavi R. (2020). Predictive model for dyslexia from fixations and saccadic eye movement events. Comput. Methods Prog. Biomed. 195:105538. doi: 10.1016/j.cmpb.2020.105538, 32526535

[ref25] Klados M. A. Styliadis C. Frantzidis C. A. Paraskevopoulos E. Bamidis P. D. (2016). Beta-band functional connectivity is reorganized in mild cognitive impairment after combined computerized physical and cognitive training. Front. Neurosci. 10:55. doi: 10.3389/fnins.2016.00055, 26973445 PMC4770438

[ref26] Klimesch W. (1999). EEG alpha and theta oscillations reflect cognitive and memory performance: a review and analysis. Brain Res. Rev. 29, 169–195. doi: 10.1016/S0165-0173(98)00056-3, 10209231

[ref27] Laine M. Salmelin R. Helenius P. Marttila R. (2000). Brain activation during Reading in deep dyslexia: an MEG study. J. Cogn. Neurosci. 12, 622–634. doi: 10.1162/089892900562381, 10936915

[ref28] Lakretz Y. Chechik G. Friedmann N. Rosen-Zvi M., “Probabilistic graphical models of dyslexia,” Proceedings of the 25th ACM SIGKDD International Conference on Knowledge Discovery & Data Mining. New York, NY, USA: Association for Computing Machinery. 1919–1928, (2015).

[ref29] Lasefr Z. Elleithy K. Reddy R. R. Abdelfattah E. Faezipour M. (2023). An epileptic seizure detection technique using EEG signals with mobile application development. Appl. Sci. 13:9571. doi: 10.3390/app13179571

[ref30] Lawhern V. J. Solon A. J. Waytowich N. R. Gordon S. M. Hung C. P. Lance B. J. (2018). EEGNet: a compact convolutional neural network for EEG-based brain-computer interfaces. J. Neural Eng. 15:056013. doi: 10.1088/1741-2552/aace8c, 29932424

[ref31] Li H. Ding M. Zhang R. Xiu C. (2022). Motor imagery EEG classification algorithm based on CNN-LSTM feature fusion network. Biomed. Signal Process. Control 72:103342. doi: 10.1016/j.bspc.2021.103342

[ref32] Liu Y. Yang F. Wu B. (2024). Compression of EEG signals with the LSTM-autoencoder via domain adaptation approach. Comput. Methods Biomech. Biomed. Engin. 1–14. doi: 10.1080/10255842.2024.2346356, 38686789

[ref33] Lizarazu M. Lallier M. Molinaro N. (2019). Phase-amplitude coupling between theta and gamma oscillations adapts to speech rate. Ann. N. Y. Acad. Sci. 1453, 140–152. doi: 10.1111/nyas.14099, 31020680 PMC6850406

[ref34] Lr H. Sudha Sadasivam G. (2022). Multimodal screening for dyslexia using anatomical and functional MRI data. J. Comput. Methods Sci. Eng. 22, 1105–1116. doi: 10.3233/JCM-225999

[ref35] Mahmoodin Z. Mansor W. Lee K. Y. Zainuddin A. Z. A., “Electroencephalogram theta-beta band power features generated from writing for the classification of dyslexic chidren,” 2018 IEEE-EMBS Conference on Biomedical Engineering and Sciences (IECBES 2018). 288–292. Sarawak, Malaysia: IEEE. (2019).

[ref36] Manghirmalani P. Panthaky Z. Jain K. (2011). “Learning disability diagnosis and classification - A soft computing approach,” in 2011 World Congress on Information and Communication Technologies, Mumbai, India: IEEE. 479–484.

[ref37] Mittag M. Larson E. Taulu S. Clarke M. Kuhl P. K. (2022). Reduced Theta sampling in infants at risk for dyslexia across the sensitive period of native phoneme learning. Int. J. Environ. Res. Public Health 19:1180. doi: 10.3390/ijerph19031180, 35162202 PMC8835181

[ref38] Mohamad N. B. Lee K. Y. Mansor W. Mahmoodin Z. Fadzal C. W. N. F. C. W. Amirin S., “EEG-based time and spatial interpretation of activation areas for relaxation and words writing between poor and capable dyslexic children,” Annual International Conference of the IEEE Engineering in Medicine and Biology Society, vol. 2015, pp. 4757–4760, Milan, Italy: IEEE. (2015). 10.1109/EMBC.2015.7319457 26737357

[ref39] Narsimha Reddy C. H. Mahesh S. Manjunathachari K. (2024). Hybrid feature integration model and adaptive transformer approach for emotion recognition with EEG signals. Comput. Methods Biomech. Biomed. Engin. 27, 1610–1632. doi: 10.1080/10255842.2023.2252551, 37688466

[ref40] Opěla P. Schindler I. Kawulok P. Kawulok R. Rusz S. Sauer M. (2022). Shallow and deep learning of an artificial neural network model describing a hot flow stress evolution: A comparative study. Mater. Des. 220:110880. doi: 10.1016/j.matdes.2022.110880

[ref41] Ortiz A. Martinez-Murcia F. J. Luque J. L. Giménez A. Morales-Ortega R. Ortega J. (2020). Dyslexia diagnosis by EEG temporal and spectral descriptors: an anomaly detection approach. Int. J. Neural Syst. 30, 2050029–2050019. doi: 10.1142/S012906572050029X, 32496139

[ref42] Parmar S. Paunwala C. (2023a). Early detection of dyslexia based on EEG with novel predictor extraction and selection. Discov. Artif. Intell. 3:33. doi: 10.1007/s44163-023-00082-4

[ref43] Parmar S. Paunwala C. (2023b). A novel and efficient wavelet scattering transform approach for primitive-stage dyslexia-detection using electroencephalogram signals. Healthc. Anal. 3:100194. doi: 10.1016/j.health.2023.100194

[ref44] Paulesu E. Frith U. Snowling M. Gallagher A. Morton J. Frackowiak R. S. . (1996). Is developmental dyslexia a disconnection syndrome?: evidence from PET scanning. Brain 119, 143–157. doi: 10.1093/brain/119.1.143, 8624677

[ref45] Peng P. Song Y. Yang L. Wei H. (2022). Seizure prediction in EEG signals using STFT and domain adaptation. Front. Neurosci. 15:825434. doi: 10.3389/fnins.2021.825434, 35115906 PMC8805457

[ref46] Perera H. Shiratuddin M. F. Wong K. W. Fullarton K. (2018). EEG signal analysis of writing and typing between adults with dyslexia and normal controls. Int. J. Interact. Multimed. Artif. Intell. 5:62. doi: 10.9781/ijimai.2018.04.005

[ref47] Power A. J. Mead N. Barnes L. Goswami U. (2013). Neural entrainment to rhythmic speech in children with developmental dyslexia. Front. Hum. Neurosci. 7:777. doi: 10.3389/fnhum.2013.00777, 24376407 PMC3842021

[ref48] Prabha A. J. Bhargavi R. Harish B. (2019). Predictive model for dyslexia from eye fixation events. Int. J. Eng. Adv. Technol. 9, 235–240. doi: 10.35940/ijeat.a1045.1291s319

[ref49] Raufi B. Longo L. (2022). An evaluation of the EEG alpha-to-Theta and Theta-to-alpha band ratios as indexes of mental workload. Front. Neuroinform. 16:861967. doi: 10.3389/fninf.2022.861967, 35651718 PMC9149374

[ref50] Ray W. J. Cole H. W. (1985). EEG alpha activity reflects attentional demands, and beta activity reflects emotional and cognitive processes. Science 228, 750–752. doi: 10.1126/science.3992243, 3992243

[ref51] Remeseiro B. Bolon-Canedo V. (2019). A review of feature selection methods in medical applications. Comput. Biol. Med. 112:103375. doi: 10.1016/j.compbiomed.2019.103375, 31382212

[ref52] Ribeiro M. H. D. M. da Silva R. G. Moreno S. R. Mariani V. C. dos Coelho L. S. (2022). Efficient bootstrap stacking ensemble learning model applied to wind power generation forecasting. Int. J. Electr. Power Energy Syst. 136:107712. doi: 10.1016/J.IJEPES.2021.107712

[ref53] Rufener K. S. Zaehle T. (2021). “Chapter 9 - dysfunctional auditory gamma oscillations in developmental dyslexia: a potential target for a tACS-based intervention,” in Non-invasive Brain Stimulation (NIBS) in Neurodevelopmental Disorders, eds. Kadosh R. C. Zaehle T. Krauel K., vol. 264 (Amsterdam, Netherlands: Elsevier), 211–232. 10.1016/bs.pbr.2021.01.016 34167657

[ref54] Safi M. S. Safi S. M. M. (2021). Early detection of Alzheimer’s disease from EEG signals using Hjorth parameters. Biomed. Signal Process. Control 65:102338. doi: 10.1016/j.bspc.2020.102338

[ref55] Shirly G. Jerritta S. (2021). Time domain analysis of electroencephalogram (EEG) signals for word level comprehension in deaf graduates with congenital and acquired hearing loss. IOP Conf. Ser. Mater. Sci. Eng. 1070:012083. doi: 10.1088/1757-899x/1070/1/012083

[ref56] Sihvonen A. J. Virtala P. Thiede A. Laasonen M. Kujala T. (2021). Structural white matter connectometry of reading and dyslexia. NeuroImage 241:118411. doi: 10.1016/j.neuroimage.2021.118411, 34293464

[ref57] Song Y. Zheng Q. Liu B. Gao X. (2023). EEG conformer: convolutional transformer for EEG decoding and visualization. IEEE Trans. Neural Syst. Rehabil. Eng. 31, 710–719. doi: 10.1109/TNSRE.2022.3230250 37015413

[ref58] Subasi A. (2007). EEG signal classification using wavelet feature extraction and a mixture of expert model. Expert Syst. Appl. 32, 1084–1093. doi: 10.1016/j.eswa.2006.02.005

[ref59] Sun H. Ye E. Paixao L. Ganglberger W. Chu C. J. Zhang C. . (2023). The sleep and wake electroencephalogram over the lifespan. Neurobiol. Aging 124, 60–70. doi: 10.1016/j.neurobiolaging.2023.01.006, 36739622 PMC9957961

[ref60] Tamboer P. Vorst H. C. M. Ghebreab S. Scholte H. S. (2016). Machine learning and dyslexia: classification of individual structural neuro-imaging scans of students with and without dyslexia. Neuroimage Clin. 11, 508–514. doi: 10.1016/j.nicl.2016.03.014, 27114899 PMC4832088

[ref61] Tawhid M. N. A. Siuly S. Wang K. Wang H. (2024). GENet: a generic neural network for detecting various neurological disorders from EEG. IEEE Trans. Cogn. Dev. Syst. 16, 1829–1842. doi: 10.1109/TCDS.2024.3386364

[ref62] Urbanowicz R. J. Meeker M. La Cava W. Olson R. S. Moore J. H. (2018). Relief-based feature selection: introduction and review. J. Biomed. Inform. 85, 189–203. doi: 10.1016/j.jbi.2018.07.014, 30031057 PMC6299836

[ref63] Wang J. Cheng S. Tian J. Gao Y. (2023). A 2D CNN-LSTM hybrid algorithm using time series segments of EEG data for motor imagery classification. Biomed. Signal Process. Control 83:104627. doi: 10.1016/j.bspc.2023.104627

[ref64] Wang H. Wu Z. Xing E. P. (2019). Removing confounding factors associated weights in deep neural networks improves the prediction accuracy for healthcare applications. Pacific Symp. Biocomput. 24, 54–65. doi: 10.1142/9789813279827_0006, 30864310 PMC6417810

[ref65] Xu Y. Yu Z. Li Y. Liu Y. Li Y. Wang Y. (2024). Autism spectrum disorder diagnosis with EEG signals using time series maps of brain functional connectivity and a combined CNN–LSTM model. Comput. Methods Prog. Biomed. 250:108196-1–108196-12. doi: 10.1016/j.cmpb.2024.108196, 38678958

[ref66] Zahia S. Garcia-Zapirain B. Saralegui I. Fernandez-Ruanova B. (2020). Dyslexia detection using 3D convolutional neural networks and functional magnetic resonance imaging. Comput. Methods Prog. Biomed. 197:105726. doi: 10.1016/j.cmpb.2020.105726, 32916543

[ref67] Zainuddin A. Z. A. Lee K. Y. Mansor W. Mahmoodin Z., “Extreme learning machine for distinction of EEG signal pattern of dyslexic children in writing,” 2018 IEEE-EMBS Conference on Biomedical Engineering and Sciences (IECBES 2018). 3, pp. 652–656, Sarawak, Malaysia: IEEE. (2019). 10.1109/EMBC.2019.8857569 31946868

[ref68] Zainuddin A. Z. A. Mansor W. Lee K. Y. Mahmoodin Z. (2022). Machine learning and deep learning performance in classifying dyslexic children’s electroencephalogram during writing. Int. J. Electr. Comput. Eng. 12, 6614–6624. doi: 10.11591/ijece.v12i6.pp6614-6624

[ref69] Zaree M. Mohebbi M. Rostami R. (2023). Biomedical signal processing and control an ensemble-based machine learning technique for dyslexia detection during a visual continuous performance task. Biomed. Signal Process. Control 86:105224. doi: 10.1016/j.bspc.2023.105224

[ref70] Zhao X. Zhang H. Zhu G. You F. Kuang S. Sun L. (2019). A multi-branch 3D convolutional neural network for EEG-based motor imagery classification. IEEE Trans. Neural Syst. Rehabil. Eng. 27, 2164–2177. doi: 10.1109/TNSRE.2019.2938295, 31478864

