# Supplementary material for: Discrete wavelet transform-driven optimized deep learning-based framework for dyslexia detection using EEG signals
Source: Front Neuroinform. 2026 Mar 25;20:1765088. doi: 10.3389/fninf.2026.1765088 (PMC13057293; doi:10.3389/fninf.2026.1765088)
Supplement: Supplementary file 5 [file Table_1.DOCX]

**Supplementary Material**

1. **Supplementary Tables**

Tables below present Layer-by-layer architecture specifications of the raw EEG end-to-end baselines (1D-CNN, Fast-LSTM, EEGNet) used for comparison with the proposed DWT feature–based DNN.

**A. 1D-CNN architecture (raw EEG)**

The 1D-CNN model takes input shape (2560, 16) and consists of three convolutional blocks followed by global average pooling and a small fully connected block:

Input: 10-s epoch, shape (2560, 16) (T×C)

**Training:** Adam (lr = 1e−3), binary cross-entropy.

| **Stage** | **Layer** | **Key configuration** | **Output shape** |
| --- | --- | --- | --- |
| Input | Input Layer | shape=(2560, 16) | (2560, 16) |
| Block 1 | Conv1D | filters=32, kernel=7, padding=same | (2560, 32) |
|  | Batch Norm + ReLU | – | (2560, 32) |
|  | MaxPool1D | pool=2 | (1280, 32) |
|  | Dropout | rate=0.3 | (1280, 32) |
| Block 2 | Conv1D | filters=64, kernel=7, padding=same | (1280, 64) |
|  | Batch Norm + ReLU | – | (1280, 64) |
|  | MaxPool1D | pool=2 | (640, 64) |
|  | Dropout | rate=0.3 | (640, 64) |
| Block 3 | Conv1D | filters=128, kernel=7, padding=same | (640, 128) |
|  | Batch Norm + ReLU | – | (640, 128) |
| Pool | GlobalAvgPool1D | – | (128) |
| FC | Dense | units=64 | (64) |
|  | Batch Norm + ReLU | – | (64) |
|  | Dropout | rate=0.3 | (64) |
| Output | Dense | units=1, activation=sigmoid | (1) |

**B. LSTM architecture (raw EEG)**

To reduce the computational cost of recurrent processing on long sequences, the time axis was down sampled prior to the LSTM layer.

Input: 10-s epoch, shape (2560, 16) (T×C)

**Training:** Adam (lr = 1e−3), binary cross-entropy

| **Stage** | **Layer** | **Key configuration** | **Output shape** |
| --- | --- | --- | --- |
| Input | Input | shape=(2560, 16) | (2560, 16) |
| Downsample | Conv1D | filters=32, kernel=7, stride=2, padding=same | (1280, 32) |
|  | BatchNorm + ReLU | – | (1280, 32) |
|  | MaxPool1D | pool=2 | (640, 32) |
| RNN | LSTM | units=32, return_sequences=False, dropout=0.2 | (32) |
| FC | BatchNorm | – | (32) |
|  | Dense | units=32 | (32) |
|  | BatchNorm + ReLU | – | (32) |
|  | Dropout | rate=0.2 | (32) |
| Output | Dense | units=1, activation=sigmoid | (1) |

**C. EEGNet architecture (raw EEG)**

EEGNet was implemented using the canonical EEGNet-style blocks (temporal convolution, depth wise spatial convolution, and separable convolution). Input epochs were reshaped to (channels, samples, 1) = (16, 2560, 1).

**Input:** **(16, 2560, 1)** (C×T×1)

| **Stage** | **Layer** | **Key configuration** | **Output shape** |
| --- | --- | --- | --- |
| Input | Input | shape=(16, 2560, 1) | (16, 2560, 1) |
| Temporal conv | Conv2D | F1=8, kernel=(1,64), padding=same, use_bias=False | (16, 2560, 8) |
|  | Batch Norm | – | (16, 2560, 8) |
| Spatial filtering | DepthwiseConv2D | kernel=(16,1), depth_multiplier D=2, padding=valid, use_bias=False | (1, 2560, 16) |
|  | Batch Norm + ELU | – | (1, 2560, 16) |
|  | AvgPool2D | pool=(1,4) | (1, 640, 16) |
|  | Dropout | rate=0.5 | (1, 640, 16) |
| Separable block | SeparableConv2D | F2=16, kernel=(1,16), padding=same, use_bias=False | (1, 640, 16) |
|  | Batch Norm + ELU | – | (1, 640, 16) |
|  | AvgPool2D | pool=(1,8) | (1, 80, 16) |
|  | Dropout | rate=0.5 | (1, 80, 16) |
| Classifier | Flatten | – | (1280) |
| Output | Dense | units=1, activation=sigmoid | (1) |

**Supplementary Note:** End-to-end learning from raw EEG is attractive for deployment, but in our dataset the raw baselines were consistently below the DWT feature-based DNN results in Table 8, suggesting that wavelet-domain features provide better sample efficiency under limited-data conditions.
